# Direct optical patterning of perovskite nanocrystals with ligand cross-linkers

**DOI:** 10.1126/sciadv.abm8433

**Published:** 2022-03-16

**Authors:** Dan Liu, Kangkang Weng, Shaoyong Lu, Fu Li, Hannikezi Abudukeremu, Lipeng Zhang, Yuchen Yang, Junyang Hou, Hengwei Qiu, Zhong Fu, Xiyu Luo, Lian Duan, Youyu Zhang, Hao Zhang, Jinghong Li

**Affiliations:** 1 Key Laboratory of Chemical Biology and Traditional Chinese Medicine Research, Ministry of Education, College of Chemistry and Chemical Engineering, Hunan Normal University, Changsha 410081, China.; 2 Department of Chemistry, Tsinghua University, Beijing 100084, China.; 3 Center for BioAnalytical Chemistry, Key Laboratory of Bioorganic Phosphorus Chemistry and Chemical Biology, Ministry of Education, Tsinghua University, Beijing 100084, China.; 4 Key Laboratory of Organic Optoelectronics and Molecular Engineering, Ministry of Education, Tsinghua University, Beijing 100084, China.

## Abstract

Precise microscale patterning is a prerequisite to incorporate the emerging colloidal metal halide perovskite nanocrystals into advanced, integrated optoelectronic platforms for widespread technological applications. Current patterning methods suffer from some combination of limitations in patterning quality, versatility, and compatibility with the workflows of device fabrication. This work introduces the direct optical patterning of perovskite nanocrystals with ligand cross-linkers or DOPPLCER. The underlying, nonspecific cross-linking chemistry involved in DOPPLCER supports high-resolution, multicolored patterning of a broad scope of perovskite nanocrystals with their native ligands. Patterned nanocrystal films show photoluminescence (after postpatterning surface treatment), electroluminescence, and photoconductivity on par with those of conventional nonpatterned films. Prototype, pixelated light-emitting diodes show peak external quantum efficiency of 6.8% and luminance over 20,000 cd m^−2^. Both are among the highest for patterned perovskite nanocrystal devices. These results create new possibilities in the system-level integration of perovskite nanomaterials and advance their applications in various optoelectronic and photonic platforms.

## Teaser

A versatile method promises direct optical patterning of perovskite nanocrystals for building integrated optoelectronics.

## INTRODUCTION

Colloidal lead halide perovskite nanocrystals (NCs) have emerged as a versatile class of materials for various optoelectronic and photonic applications (*1*, *2*). A multitude of synthetic routes and ligand designs have endowed perovskite NCs with remarkable controllability in compositions, sizes, and surface chemistry (*3*–*5*). In particular, the surface chemistry–dominant properties distinguish NCs from perovskites in other forms, and the size-dependent exciton binding energy offers an additional degree of freedom in tuning their photophysical properties (*6*, *7*). These features promise their use in high-performance light sources and light harvesters, such as light-emitting diodes (LEDs) (*7*, *8*), solar cells (*9*, *10*), photo- and x-ray detectors (*11*), lasers (*12*), and unconventional single photon emitters (*13*). For instance, the maximum external quantum efficiency (EQE) of perovskite NC LEDs has boosted to over 20%, approaching those of quantum dot (QD) LEDs (*7*, *8*). In parallel to the advances at the material- and single-device level, it is critical to develop effective patterning methods to locate perovskite NCs at given positions with microscale precision. These patterning capabilities are prerequisites for their practical applications in system-level integrated platforms, such as full-colored high-resolution displays and multiplexed image sensors.

An ideal patterning method should be (i) generally applicable to perovskite NCs with various core properties and surface states tailored for desirable physical and chemical properties, (ii) fully compatible with workflows in building system-level devices, and (iii) capable to produce high-resolution, uniform patterns in a high-throughput manner. Unfortunately, patterning of perovskite materials, especially NCs, remains extremely challenging because of a combination of their ionic nature, labile surface chemistry, vulnerable structural integrity, and sensitive optical/electrical properties (*14*). Existing methods cannot simultaneously accomplish all the above requirements. X-ray/electron beam lithography (*15*, *16*) and laser direct writing (*17*) suffer from severe loss of photoluminescence (PL) and high propensity of NC sintering. Inkjet printing supports multicolored patterning of perovskite nanomaterials under mild condition with decent PL quantum yield (PLQY) (typically ~60%) (*18*–*21*). However, high-resolution (e.g., pixel sizes smaller than 20 μm) patterns with film quality favorable for optoelectronic devices are hard to achieve because of the complexities in ink formula and in controlling fluidic dynamics at micrometer scale. Advanced micro- and nanoprinting techniques render ultrahigh resolution patterning of perovskite NCs but require sophisticated apparatus and prepatterned substrates (*22*, *23*). In comparison, photolithography represents a cost-effective route for generating high-resolution patterns in a parallel fashion (*24*). Unfortunately, the ionic nature renders perovskite NCs incompatible with the solvents used at various stages of traditional photolithography (resist coating, developing, and lift-off). To circumvent this, modified photolithographic methods use additional photoresist layers, careful control of dry etching steps, and specific combinations of orthogonal solvents/photoresists (*24*). These procedures prevent their integration with the fabrication workflows of optoelectronic devices with multilayered structures. As a consequence, only a handful of reports demonstrate patterned electroluminescent (EL) perovskite NC LEDs, showing low EQEs (~3%) and low brightness (<10,000 cd m^−2^) (*20*, *21*).

Photoresist-free, direct optical lithography of functional inorganic nanomaterials (DOLFIN), proposed by Talapin group (*25*), represents an alternative, material-adapted patterning method for NCs. DOLFIN inherits the benefits of traditional photolithography and relies critically on the design of photoactive ligands. The light-driven ligand decomposition at NC surface introduces contrast in colloidal stability of NCs, allowing for the microscale patterning of NC thin films. The concept works well for a library of short, photosensitive ligands, various NCs, and devices (*26*, *27*). However, it typically entails ligand exchange process and polar solvents, which can structurally and/or optically damage perovskite NCs. Note that a very recent work addressed these issues by using oxime sulfonate esters as photosensitive ligands and toluene as the solvent for perovskite NCs (*28*). Complementing the design of short, photodecomposable ligands, polymeric ligands with cross-linkable fragments also allow direct patterning of perovskite NCs via cycloaddition, polymerization, and other pathways (*29*). However, patterning with these polymeric ligands usually yields NC composites in an insulating matrix with hindered charge injection/extraction/transport (*29*–*31*). In addition, these direct patterning methods for perovskite NCs require judicious selection of photoactive ligands (short/decomposable or long/cross-linkable). These selected ligands replace the native ligands and thus limit the use of well-designed core and surface chemistry for NCs toward favorable electronic and optical properties.

Here, we develop an effective method for direct optical patterning of perovskite NCs with ligand cross-linkers (termed as DOPPLCER), which does not require ligand exchange. DOPPLCER uses bisazides (nitrene precursors) as photoactive additives that cross-link adjacent NCs via covalently bonding to their native, alkyl ligands when exposed to ultraviolet (UV; 254 or 365 nm). This process substantially reduces the solubility of exposed NCs in toluene or other nonpolar solvents and yields uniform patterns with resolution approaching the limit of the photomasks (~5 μm in our case). The nonspecific photochemistry renders DOPPLCER universal and amenable to a wide variety of inorganic or hybrid organic-inorganic perovskite NCs with different cores, sizes, and surface states, supporting red green blue (RGB) multicolored patterning of NCs at low exposure doses. DOPPLCER is also compatible with different surface treatment protocols for better surface passivation or improved electronic coupling. Patterned films of CsPbBr_3_ and formamidinium lead bromide (FAPbBr_3_) NCs retain up to 60% of their initial PLQY, and postpatterning surface treatment increases their absolute PLQY to ~76%, exceeding those of pristine films. Replacing the native ligands in patterned NC thin films with more compact species yields devices with decent photoconducting behavior. DOPPLCER adapts well to the fabrication procedures for EL LEDs and allows for constructing prototype devices with pixelated, NC emissive layers. FAPbBr_3_ NC–based LEDs made via DOPPLCER show a peak EQE of ~6.8% and maximum luminescence over 20,000 cd m^−2^. Both are among the highest values reported for patterned perovskite LEDs. The combined capabilities of DOPPLCER create a versatile platform for building system-level, integrated devices of perovskite NCs, with implications in practical applications in displays, image sensors, and other advanced optoelectronic and photonics.

## RESULTS

### Description of DOPPLCER and related ligand chemistry

**Fig. 1.**
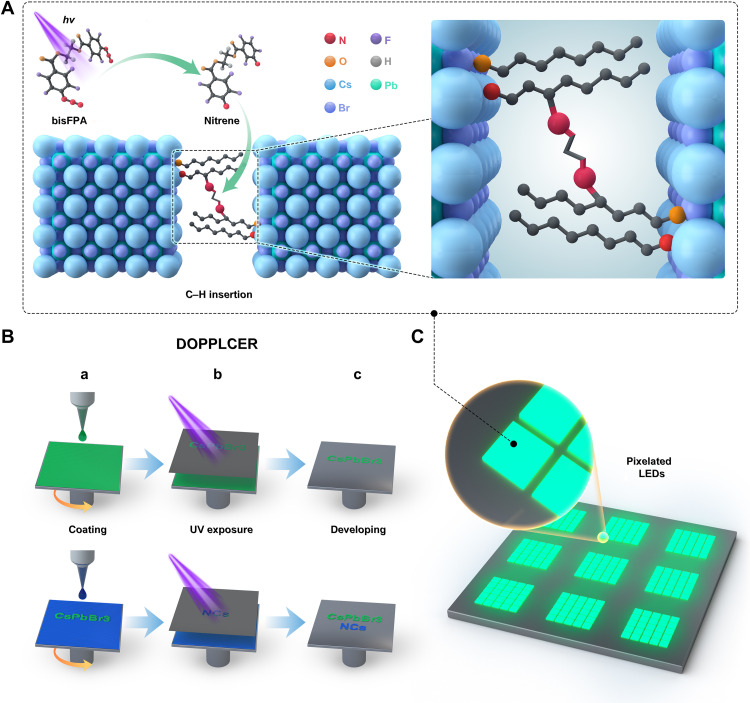
Schematic illustration of DOPPLCER. (**A**) Description of the photocrosslinking chemistry of native ligands on perovskite NCs (e.g., CsPbBr_3_). Under UV irradiation, bisFPA additives release nitrogen and generate nitrene radicals at both ends (top left) that undergo C─H insertion reaction with the native ligands on neighboring NCs (bottom left and right). This photochemistry is nonspecific and capable of linking aliphatic ligands with various anchoring groups, as represented by oleylammonium (with terminal N atom in red color) and oleate (with terminal O atom in orange) ligands. The yielded NC networks lose their colloidal dispersibility. (**B**) Outline of the procedures for DOPPLCER (top), including (a) NC coating, (b) UV exposure at selected regions, and (c) developing of NC patterns with nonpolar solvents. Repeating these steps enables sequential, multilayered, and multicolored patterning of perovskite NCs (bottom). (**C**) DOPPLCER is compatible with the workflows for constructing pixelated devices, such as LEDs with emissive NC patterns.

Figure 1 shows the concept and procedures of DOPPLCER for patterning perovskite NCs (e.g., CsPbX_3_ NCs) with their native ligands. This method relies on the photochemistry of bisazide cross-linkers added to NC inks. Under UV irradiation (254 or 365 nm, depending on the molecular design), bisazides release nitrogen and create highly reactive, singlet nitrene radicals at both ends (Fig. 1A, top left). The nitrene radicals readily form covalent C─N bonds with the long alkyl chains in the native ligands [e.g., oleylamine (OLAm) and oleic acid (OA)] via C─H insertion. These photochemical events, when occurring between ligands on neighboring NCs, lead to cross-linked NC networks insoluble in nonpolar solvents (Fig. 1A and fig. S1). The markedly altered colloidal stability sets the basis of DOPPLCER, which includes three steps (Fig. 1B, top): (i) Coating of an ink composed of NCs and bisazide additives onto substrates, (ii) UV exposure at selected regions via a predesigned photomask, and (iii) developing with a nonpolar solvent (toluene, hexane, chlorobenzene, etc.) to remove unexposed NCs. Repeating this process allows for sequential, layer-by-layer patterning of the same or different perovskite NCs that are suitable for creating multicolored patterns (Fig. 1B, bottom). The entire process eliminates the use of conventional photoresists, polar solvents, or other chemicals detrimental to perovskite NCs. The nonspecific and efficient nitrene C─H insertion allows for patterning of NCs with different core properties and surface ligands. The method also uses infrastructures and steps fully adaptable in solution-based fabrication of multilayered optoelectronic devices. The combined features of DOPPLCER support unique capabilities in building high-performance, pixelated LEDs (Fig. 1C).

The choice of bisazides dictates the efficacy of the presented patterning method. Previous reports on the photolysis of bisazides have correlated their chemical structures with kinetics in different reaction pathways (*32*). Guided by these results, we selected ethylene bis(4-azido-2,3,5,6-tetrafluorobenzoate) as an example. It is a representative bis(fluorophenyl azide) (bisFPA) photocrosslinker used in conventional negative photoresists (*33*) and, more recently, in the patterning of functional polymers (*34*) and QDs (*35*). Figure 1A and figs. S2 and S3 show details on its chemical structure, synthesis, and nuclear magnetic resonance (NMR) spectra. Consistent with previous reports (*32*), these bisFPA molecules show strong absorption in deep UV region with a maximum extinction coefficient of ~2.9 × 10^4^ cm^−1^ M^−1^ at around 260 nm (fig. S4), supporting their efficient photolysis to nitrene radicals. The perfluorophenyl backbones in bisFPA suppress side reactions, such as ring expansion to ketenimine and triplet formation of aniline or azo-type compounds, and guarantee the desirable singlet nitrene C─H insertion as the predominant pathway (*32*). For instance, a previous work found that the yields of ketenimine and triplet by-products were below 2% when using perfluorophenyl bisazides as photocrosslinkers for semiconducting polymers (*34*).

**Fig. 2.**
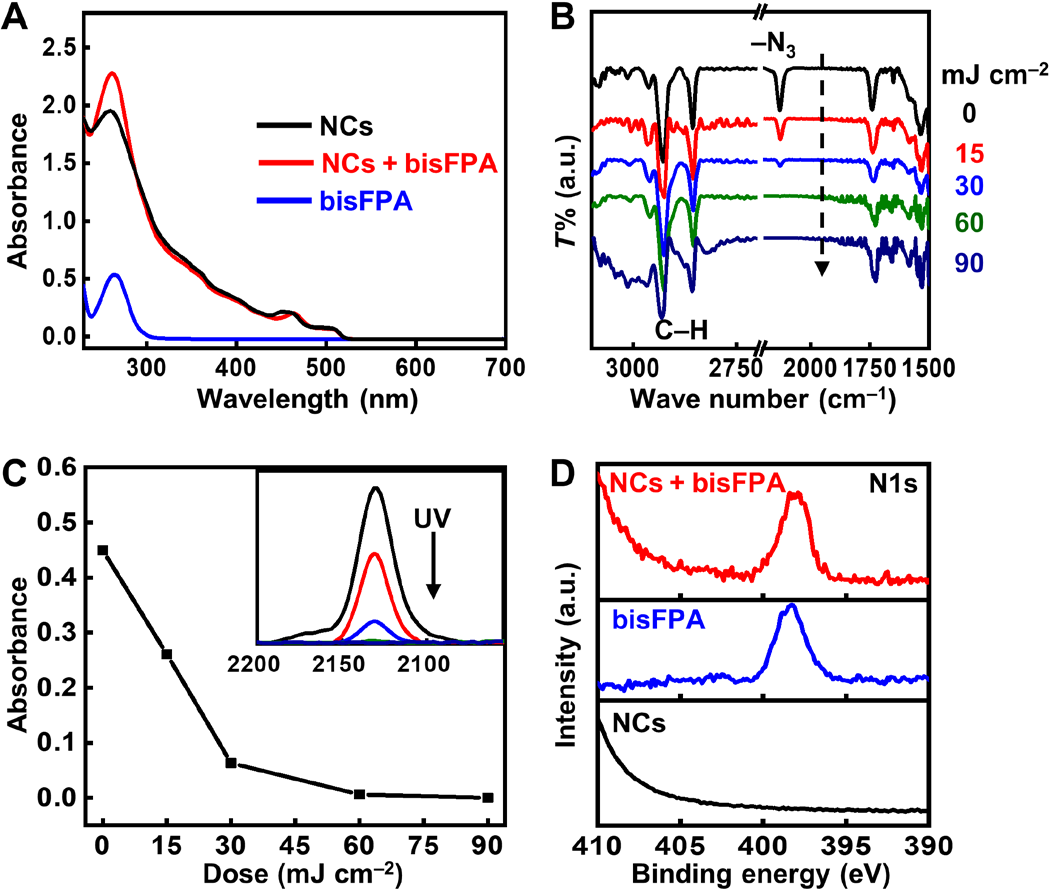
Characterizations of the ligand chemistry involved in DOPPLCER. (**A**) UV-vis spectra of solutions of CsPbBr_3_ NCs (black), bisFPA (blue), and their mixtures (red). The mass ratio of bisFPA to NCs is 20 wt%. bisFPA shows strong absorption at about 260 nm, which imposes on that of NCs. (**B**) FTIR spectra of thin films made from an ink of CsPbBr_3_ NCs and bisFPA, followed by UV irradiation (at 254 nm) with different doses (0, 15, 30, 60, and 90 mJ cm^−2^; from top to bottom). a.u., arbitrary units. (**C**) Changes in the IR absorbance of azido (─N_3_) group at 2130 cm^−1^ with prolonged UV exposure, derived from data shown in (B). Inset shows the decrease in IR absorbance with increasing UV doses (0, 15, and 30 mJ cm^−2^; from top to bottom). (**D**) XPS N1s spectra of thin films of OPA-capped CsPbBr_3_ NCs (black), bisFPA (blue), and a film of NCs with 20 wt% of bisFPA treated by DOPPLCER (red; dose of 60 mJ cm^−2^, followed by solvent developing).

The selected bisFPA, when mixed with NCs, retain their high efficiency and fast kinetics in the photochemistry involved in DOPPLCER (Fig. 2), namely, the photolysis and the subsequent C─H insertion. We used CsPbBr_3_ NCs with well-studied synthetic protocols and surface chemistry (*36*) as a model system. The surface chemistry of these NCs features a mixture of labile ligands (oleylammonium bromide and oleylammonium oleate) and residual noncoordinating octadecence (ODE) from the solvent (*37*). For simplicity, we use OLAm and OA to represent the ionic bonding species in the following discussion. UV-visible (UV-vis) absorption spectrum (Fig. 2A) and transmission electron microscopic (TEM) analysis of these CsPbBr_3_ NCs (fig. S5) show ~7.7-nm nanocubes with the first excitonic peak at ~514 nm, consistent with those in previous reports. Adding bisFPA to CsPbBr_3_ NC ink [the mass ratio of bisFPA:NCs ≈ 20 weight % (wt%)] leads to a notable increase in absorbance at around 260 nm. The comparable absorption coefficients of NCs and bisFPA (see the Supplementary Materials) support fast photolysis of bisFPA and generation of nitrene radicals in NC thin films. Fourier transform infrared (FTIR) spectra provide semiquantitative insights on this process (Fig. 2B). Unexposed thin films (black curves in Fig. 2B and fig. S6) show strong bands at ~2130 and ~1250 cm^−1^, corresponding to the asymmetric and symmetric stretching modes of azido moieties (*34*). Bands at 2800 to 3000 cm^−1^ and 1300 to 1500 cm^−1^ are characteristic for C─H vibrational modes, which arise from the native ligands (mostly OLAm and OA) of CsPbBr_3_ NCs. The features associated with ─N_3_ decrease with increasing exposure doses (at 254 nm) and disappear at doses over ~60 mJ cm^−2^. During the same period, peaks for C─H stretching modes (2800 to 3000 cm^−1^) remain largely unchanged and can serve as internal standard (fig. S6B). By normalizing the intensities of C─H stretching peaks in the absorbance mode, we observed a sharp decline (over 80%) in the ─N_3_ absorption during the course of 0 to 30 mJ cm^−2^, followed by disappearance with doses over 60 mJ cm^−2^ (Fig. 2C). Note that these exposure doses are comparable to those required for commercial photoresists and remarkably lower than those for NCs with photoactive ligands containing cross-linkable C═C and benzophenone groups (in the order of several J cm^−2^) (*30*, *31*). Patterning of perovskite NCs at low doses is critical for reducing photodamages. FTIR spectra also suggest the formation of C─N bonds as a result of the desirable nitrene C─H insertion reaction. This is evidenced by the evolution of asymmetric C─N─C peaks (~1310 cm^−1^) with increasing exposure doses (fig. S6C). However, we did not observe the symmetric C─N─C (expected at ~1250 cm^−1^) band even in the overexposed thin films (dose, >90 mJ cm^−2^). The absence of this peak might be due to its overlap with the symmetric ─N_3_ vibration and/or the low signal-to-noise ratio. To provide unambiguous evidence of C─N formation, we compared the x-ray photoelectron spectroscopy (XPS) spectra of thin films of CsPbBr_3_ NCs capped solely with octaphosphoric acid (OPA) ligands (*38*), bisFPA, and their mixtures (Fig. 2D and fig. S7). As expected, OPA-capped NCs show no features in the N1s spectra. Thin films composed of both NCs and bisFPA, after UV exposure and solvent developing to remove unreacted bisFPA, still show an evident N1s peak with binding energy similar to that of pure bisFPA. The occurrence of N1s peak supports the formation of C─N bonds during DOPPLCER that cross-links NCs. The loss of colloidal stability can be explained by the formation of large, insoluble collections of glued NCs (*15*, *39*). Alternative interpretation may be related to the limited C─C bond rotation/skeleton bending in cross-linked ligands and the reduced intramolecular entropy change during the dissolution of NC-ligand complexes (*40*).

The photochemistry described above involves reactions mainly between bisFPA and NC ligands. DOPPLCER can thus be engineered by changing the ratio between these two reactants. For instance, purification procedures of NCs strongly affect the amount of surface ligands and thus play a pivotal role in the patterning efficiency. Details about NC purification procedures denoted as (A) to (F) appear in Materials and Methods. As shown in fig. S8 (A to C), CsPbBr_3_ NCs with one cycle of purification form no patterns. In comparison, NCs with an additional purification step yield notable patterns under otherwise the same patterning condition (fig. S8, D to F). This can be traced to the changes in the amount of surface ligands in NC inks due to different purification procedures. A combination of ^1^H NMR and UV-vis absorption spectroscopy enables the quantification of the amount of ligands, nominal ligand coverage, and others (table S1). Briefly, the concentration of oleyl species, including (protonated) OLAm and OA, can be estimated from the intensity of alkene resonance at ~5.33 parts per million (ppm) in the ^1^H NMR spectra (figs. S9 and S10). The intensity of resonant peaks at 4.94 and 5.80 ppm, characteristic of a terminal alkene, provides the concentration of ODE. UV-vis absorption spectra allow the estimation of the concentration of CsPbBr_3_ NCs by using the intrinsic absorption coefficient reported elsewhere (*41*). NCs with a single cycle of purification shows high mass fraction of oleyl-based ligands (~30% of the mass of NCs) and, thus, high nominal ligand densities (e.g., 3.7 nm^−2^ for NCs after purification procedure A; table S1). Note that the calculated nominal ligand densities include ligands in both bound and free states and thus can exceed the theoretical ligand density of 2.9 nm^−2^ (*37*). These NCs also have a substantial amount of residual ODE with similar or even higher concentration compared to the ligands. As free ligands and noncoordinating ODE consume bisFPA without contributing to NC cross-linking, these NCs form no noticeable patterns. Another round of purification substantially reduces the concentration of oleyl species and ODE by several folds and about one order of magnitude, respectively. The resultant, moderate ligand density (<1 nm^−2^) and trace ODE notably suppress the “side reactions” between bisFPA and unbound species, enabling the formation of notable, high-quality NC patterns.

Film retention, defined as the percentage of NCs retained in the exposed films after developing, provides a quantitative evaluation of the patterning efficiency for NCs with different purification procedures. Purification procedures with more nonsolvent in the first cycle of wash lead to increased film retention, from 62 to 80% (fig. S10B). This increase mainly follows from the reduced content of ODE (table S1). The rapid increase in film retention also confirms the fast kinetics of photochemistry in DOPPLCER. A low dose of 15 mJ cm^−2^ results in over 60% film retention that subsequently saturates (~80%) at 60 mJ cm^−2^. This trend is consistent with the kinetics of bisFPA decomposition shown by FTIR analysis (Fig. 2C). The nonzero film retention for unexposed thin films may come from residual NCs after solvent developing, which was also observed in previous reports on direct optical patterning of QDs (*25*, *26*). The measured film retention maximum (based on inductively coupled plasma-optical emission spectroscopy analysis of Pb atoms) does not approach 100%, partially because of the loss of Pb-containing species in the forms of NC films detached from the substrates and/or labile Pb-ligand complexes desorbed from NC surface. The amount of bisFPA, the other component in the photocrosslinking chemistry, also determines the patterning quality (fig. S11). Five weight percent and higher contents of bisFPA are sufficient to pattern thin film of CsPbBr_3_ NCs (ligand density, <1 nm^−2^). High-quality patterned films of CsPbBr_3_ and FAPbBr_3_ NCs for optical and electrical measurements (as discussed below) can be obtained by adding 10 to 20 wt% of bisFPA molecules. In the case of 10 wt% of bisFPA, the molar ratio between bisFPA and oleyl ligands (purification procedure F: ligand density, ~0.6 nm^−2^) is around 2:1. For reference, the theoretical cross-linking threshold for polymers requires twofolds of cross-linkers with respect to the number of polymer chains to achieve film retention over 60% (*34*). Considering that a sizable fraction of cross-linking in DOPPLCER occurs between ligands on the same NC and multiple cross-linking events between the same pair of ligands, the overall patterning efficiency for DOPPLCER is high. This high patterning efficiency, originated from the nonspecific nature of nitrene insertion, distinguishes DOPPLCER from methods based on ligands with specific, cross-linkable fragments. The latter typically involve high mass ratio (ligand/NCs over 100%) of long, insulating polymeric ligands (*29*, *31*) and high exposure doses (*31*). Similar contrast in patterning efficiency of the nonspecific and specific cross-linking mechanism has been observed in the case of polymers (*34*).

### High-resolution, multicolored patterning of perovskite NCs

**Fig. 3.**
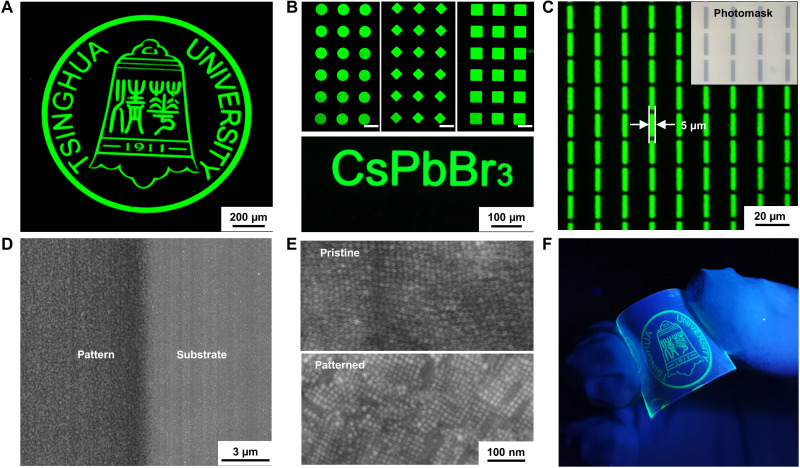
Optical and microscopic images of CsPbBr_3_ NC patterns obtained via DOPPLCER. (**A** to **C**) Fluorescence optical microscopic images of CsPbBr_3_ NC patterns in the formats of Logo, microdot/diamond/square arrays, and letters. Scale bars, 100 μm [(B), top]. Inset in (C) shows the bright-field optical image of the photomask used to obtain the rectangular arrays. (**D**) SEM image of a photopatterned CsPbBr_3_ NC thin film magnified at the edge, showing the difference between exposed and unexposed parts. (**E**) Top-view SEM images of (top) pristine and (bottom) patterned NC thin films, both featuring densely packed cuboid NCs. (**F**) A photograph of a patterned NC thin film on a flexible substrate, taken under UV light. Photo credit: Dan Liu and Hengwei Qiu, Tsinghua University.

The design concept and parameter control of DOPPLCER allow fast, high-resolution, and high-fidelity patterning of inorganic and hybrid organic-inorganic perovskite NCs passivated with their native ligands. Using green-emitting CsPbBr_3_ NCs with OLAm/OA ligands as an example, Fig. 3 (A to C) shows fluorescence microscopic images of patterned NC films in the formats of complex logo, microdot/diamond/triangle arrays, letters, and lines. The width of rectangular patterns in Fig. 3C is 5 μm, replicating those of the predesigned photomasks. The height profile of the line patterns (fig. S12) shows the high uniformity of these patterns with film thickness of ~30 nm and sharp edges. The film thickness can be tuned by changing the concentration of NC inks and the coating parameters. Scanning electron microscopic (SEM) image in Fig. 3D also reveals a marked contrast between the exposed and unexposed regions. The mild condition used in DOPPLCER allows CsPbBr_3_ NCs to retain their morphological properties. Top-view SEM images in Fig. 3E compare the morphology of pristine and patterned CsPbBr_3_ NC thin films, both featuring densely packed NC cuboids with similar uniformity and surface roughness. The sizes and orthorhombic crystal structures of NCs remain unchanged after patterning, as indicated by both SEM images (Fig. 3E) and x-ray diffraction patterns (fig. S13). Sintering or necking of NCs, which typically occurred in laser writing approach (*17*), is not observed here. The combined features of high resolution, high fidelity, simplicity, and mild condition render DOPPLCER a reliable patterning approach that applies well to both rigid and flexible substrates (Fig. 3F).

**Fig. 4.**
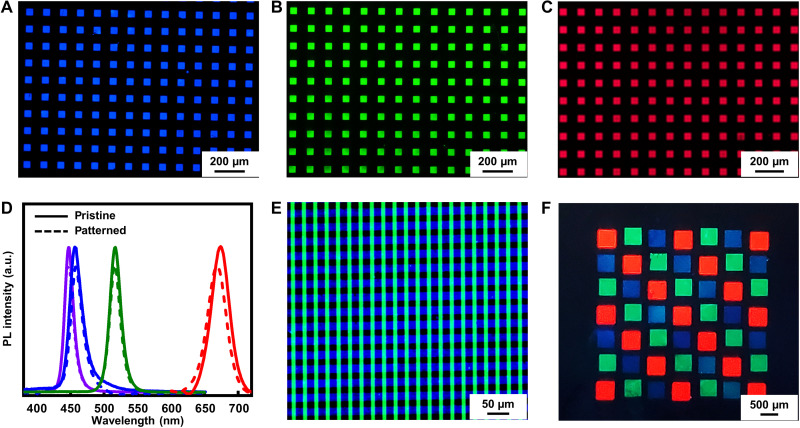
Multicolored patterning of various perovskite NCs via DOPPLCER. Fluorescence optical microscopic images of patterns of (**A**) blue-emitting CsPbBr_3_ QDs, (**B**) green-emitting FAPbBr_3_ NCs synthesized by LARP, and (**C**) CsPbI_3−*x*_Br*_x_* NCs by anion exchange of CsPbBr_3_ NC patterns with iodide. (**D**) PL spectra of pristine (solid) and patterned (dashed) thin films of CsPbCl_3−*x*_Br*_x_* NCs (violet), CsPbBr_3_ QDs (blue), CsPbBr_3_ NCs (green), and presynthesized CsPbI_3−*x*_Br*_x_* NCs (red), respectively. (**E**) Fluorescence optical microscopic images of two-color stacked line patterns made of blue-emitting CsPbBr_3_ QDs and green-emitting CsPbBr_3_ NCs. (**F**) A photograph of RGB patterns of QDs (red), CsPbBr_3_ NCs (green), and CsPbBr_3_ QDs (blue), taken under UV light.

DOPPLCER is universal to perovskite NCs with various compositions, sizes, and surface chemistry and synthesized by different methods (Fig. 4). TEM images and UV-vis absorption spectra of some of perovskite NCs used in this work appear in fig. S14. Besides NCs with labile OLAm/OA ligands, CsPbBr_3_ NCs capped with tightly bound phosphoric acid (*38*) or zwitterionic ligands [3-(*N*,*N*-dimethyloctadecylammonio) propanesulfonate] (*42*) can form patterns following similar procedures (fig. S15). The versatility in ligand designs allows NC patterns to incorporate the rich toolbox of surface chemistry developed for improved optical and electrical properties. DOPPLCER also works well for inorganic and organic/inorganic hybrid perovskite NCs synthesized by ligand-assisted reprecipitation (LARP) method (*43*), as exemplified by FAPbBr_3_ NCs (*44*) (Fig. 4B). This greatly expands the scope of applicable perovskite NCs. DOPPLCER can be further extended to NCs with different emission colors in a widely tunable range. First, the preservation of native ligand shell and mild condition encourage the patterning of NCs with sizes in quantum confinement regime and size-dependent emission wavelengths. For instance, thin films of 3.9-nm CsPbBr_3_ QDs retain their size-defined, blue emission before and after patterning (Fig. 4A). CsPbCl_3−*x*_Br*_x_* with tunable halide compositions can also form blue-emitting patterns, whose emission profiles remain identical to those of original NCs (Fig. 4D). Similarly, red-emitting CsPbI_3−*x*_Br*_x_* NCs can be patterned via DOPPLCER. However, these patterned NCs show notably reduced PL emission, presumably due to their intrinsic instability. In comparison, postpatterning anion exchange (*45*) with a combination of iodide salts (PbI_2_ or trimethylsilyl iodide) and OLAm/OA converts CsPbBr_3_ NCs to CsPbI_3−*x*_Br*_x_* patterns with bright, red emission (Fig. 4C). The facile postpatterning anion exchange reaction suggests that the patterned NC cores remain reactive and accessible after photocrosslinking of their native ligands. This is in stark contrast with previous reports on perovskite NCs with cross-linked ligands via x-ray irradiation or with polymeric ligands containing cross-linkable fragments. In those cases, the dense, cross-linked ligand shells/matrices prevent the transformation of perovskite NCs in the presence of halides or polar environments (*16*, *29*). This difference suggests that DOPPLCER uses a small amount of ligands and induces low degree of cross-linking. Cross-linking of native ligands at a moderate level without introducing more barriers for charge transport is critical for the performance of patterned optoelectronic devices, as described below. Figure 4D summarizes the emission profiles of these NC patterns, highlighting the capability of DOPPLCER in patterning NCs with desirable composition and optical properties. The mild condition used in DOPPLCER aids the preservation of optical absorption (fig. S16) and emission characteristics before and after cross-linking.

The versatile DOPPLCER method also enables multicolored patterning of perovskite NCs. Previous lithographic methods for perovskites in NC and other forms rely on special photoresists, additional protective layers and multiple dry-etching steps, which complicate the multicolored patterning process. In comparison, DOPPLCER can realize multicolored patterns with much fewer steps, as shown in Fig. 1B, aided by proper choices of NCs. Note that anion exchange reaction readily occurs when NCs with different anions are in physical contact during the multilayered patterning process. This was also observed in a recent report on perovskite NC patterning with photocleavable ligands (*28*). As DOPPLCER is generally applicable to various NCs, this issue can be addressed by using NCs with the same anion but different emission colors. Figure 4E shows consecutively patterned, stacked lines of CsPbBr_3_ NCs with different sizes and, thus, emission wavelengths in blue and green region, respectively. The sharp line edges (width, ~10 μm) and unaltered colors confirm minimal interference (e.g., redissolution of the underlying layer) during the sequential patterning of these two layers. Full-colored, RGB patterns can be made with an additive layer of red-emitting QDs composed of II-VI compounds (Fig. 4F). Figure S17 shows pixelated RGB patterns made from the same combination of materials, where the pixel size is about 10 μm by 50 μm, corresponding to ~400 pixels per inch. RGB patterns made solely with perovskite NCs can be potentially obtained by replacing II-VI QDs with recently reported, red-emitting CsMnBr_3_ NCs (*46*) with decent PLQY and structural stability. Other strategies include coating perovskite NCs with dense polymer layers/inorganic shells or introducing intermediate layer of poly(methyl methacrylate) between two consecutive perovskite NC layers; both substantially suppress anion exchange reactions.

### PL, EL, and photoconductive properties of patterned perovskite NCs

**Fig. 5.**
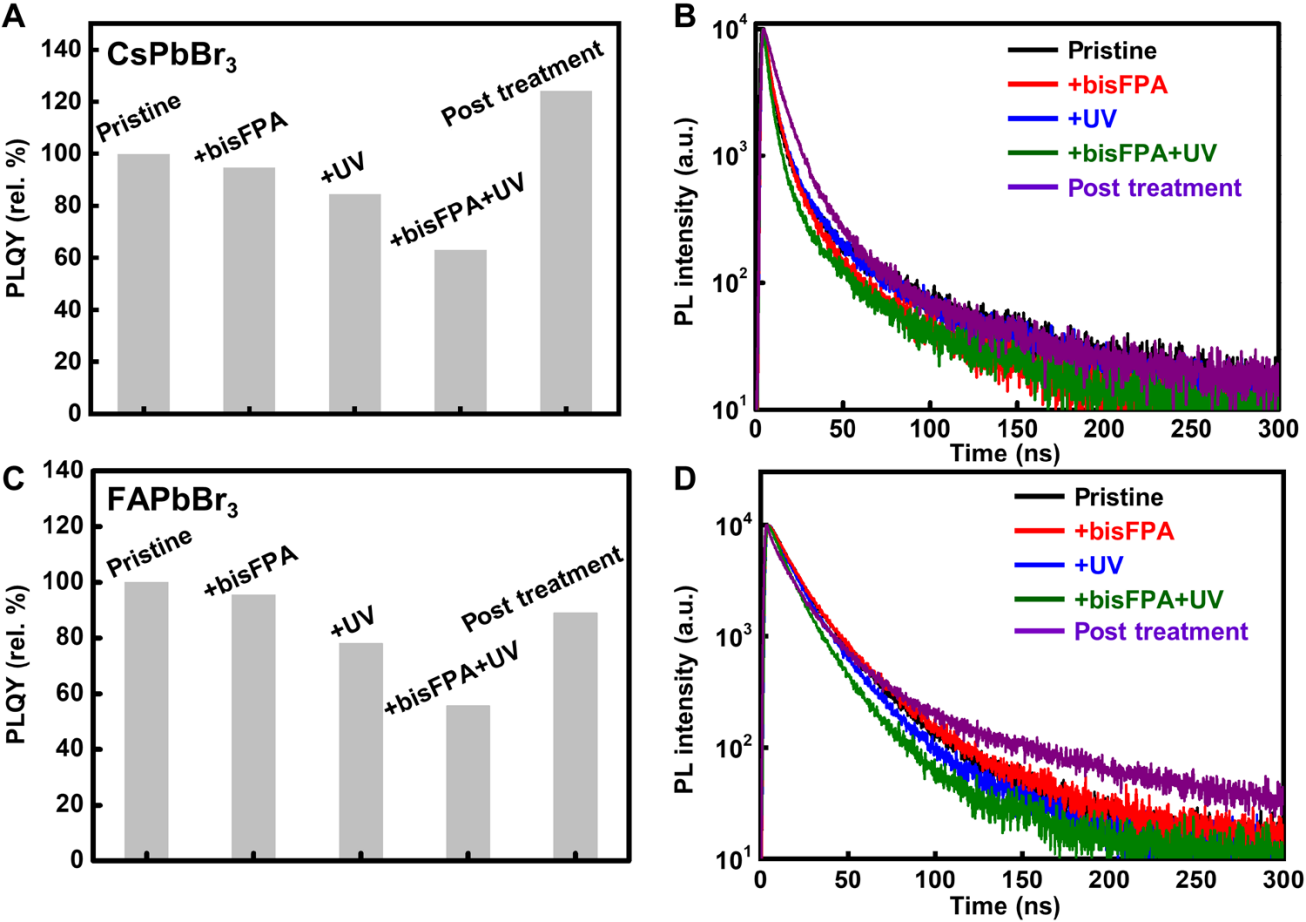
PL characteristics of perovskite NCs during DOPPLCER. Relative PLQY of thin films of (**A**) CsPbBr_3_ and (**C**) FAPbBr_3_ NCs with different treatments involved in DOPPLCER, including pristine films, films with bisFPA additives (+bisFPA), UV exposed (+UV), UV exposed in the presence of bisFPA (+bisFPA+UV), and patterned films with posttreatment (brief soaking in a mixture of PbBr_2_/OLAm/OA in ethyl acetate). (**B** and **D**) Corresponding PL decay curves of film samples in (A) and (C), respectively. Samples shown in (A) and (C) are prepared under conditions used in DOPPLCER, namely, 10 or 20 wt% of bisFPA and UV dose of 60 mJ cm^−2^.

Nondestructive NC patterning methods are desirable for high-performance, pixelated optoelectronic devices. The effect of DOPPLCER on the optical properties of perovskite NCs was studied by measuring steady-state and time-resolved PL spectra and monitoring the PLQY of NCs at various patterning steps. As shown in Fig. 4D, the peak position and width of PL spectra remain the same for NC thin films before and after patterning under DOPPLCER condition, namely, with 10 to 20 wt% bisFPA and a UV dose of 60 mJ cm^−2^ (254 nm). This suggests that the photochemistry in DOPPLCER does not affect the composition or electronic structure of perovskite NCs. We also monitored the changes in PLQY of NC thin films at different patterning steps. Pristine thin films composed of purified CsPbBr_3_ NCs with OLAm and OA ligands serve as the model system. PLQY of these thin films is 61% (Fig. 5A), consistent with reported values (*47*). Adding 20 wt% bisFPA introduces a negligible decrease in PLQY (from ~60 to 58%; relative remnant PLQY, >95%; Fig. 5A and table S2). UV exposure at 254 nm results in slightly more reduction in PLQY (remnant PLQY, ~85%). In comparison, CsPbBr_3_ NC thin films treated under DOPPLCER condition, i.e., both the addition of bisFPA and UV exposure of 60 mJ cm^−2^, show a notable decrease in PLQY to 63% of their original value. To explore the origin of the decreased PLQY, we estimated the highest occupied molecular orbital (HOMO; 7.0 eV versus vacuum) and lowest unoccupied molecular orbital (LUMO; 2.9 eV) of UV-exposed bisFPA molecules by using UV photoelectron spectroscopy (fig. S18). Details appear in the Supplementary Materials. The HOMO and LUMO of bisFPA lie outside the conduction band minimum and valence band maximum of CsPbBr_3_ NCs. This benign electronic energy level alignment suggests that bisFPA may not act as carrier or exciton traps, which explains the negligible decrease in PLQY after bisFPA addition. The decreased PLQY under DOPPLCER condition is then attributed to the side reactions of photogenerated nitrene with atoms at NC surface and the associated trap states (*35*). These assumptions, however, need to be verified with further studies. Nonetheless, films after DOPPLCER still maintain an absolute PLQY of 38%, which compares favorably with those patterned by photocleavable oxime sulfonate ester ligands (PLQY, <5%) (*28*). Shorter averaged lifetime from time-resolved PL measurements (Fig. 5B and table S3) correlates with lower PLQY and supports the assumption of increased nonradiative decay rates. Films with bisFPA additives or UV exposure show slightly reduced average lifetime (~6.6 and 7.1 ns, respectively, versus 7.5 ns for pristine films), while those treated under DOPPLCER condition show notably shorter average lifetime of 4.6 ns. Similar trend was observed for FAPbBr_3_ NCs (Fig. 5, C and D, and table S4). The absolute PLQYs (remnant PLQYs shown in parenthesis) of pristine, bisFPA (10 wt%)–added, UV-exposed (60 mJ cm^−2^ at 254 nm), and DOPPLCER-treated FAPbBr_3_ NC films are 86%, 82% (96%), 67% (78%), and 48% (56%), respectively. Several strategies may be used to achieve higher remnant PLQY in DOPPLCER. Surface treatment strategies, proven to be effective in increasing the PLQY of perovskite NCs in solution and film states (*48*), support remarkable recovery of PLQY of NC thin films after DOPPLCER. For example, a brief soaking of DOPPLCER-treated CsPbBr_3_ NC thin films in a mixture of PbBr_2_/OLAm/OA in ethyl acetate results in a twofold increase in absolute PLQY from 38 to 76% (Fig. 5A), exceeding that of the pristine NC thin films (61%). The increased PLQY can be attributed to improved surface passivation with additional Br anions introduced during surface treatment. This argument is supported by the higher Br/Pb ratio (increased by 10%) revealed by XPS analysis (fig. S19) and longer average PL lifetime of ~10.2 ns (table S3), characteristic for better surface passivation. The lower Br contents in patterned NCs might be related to the undesirable reactions between photogenerated nitrene radicals and surface Br atoms. Evidence on the formation of related produces is needed to support this presumption. In addition, it is somewhat unexpected to observe the disappearance of F1s signals after this surface treatment (fig. S19, C and F), suggesting that the moieties of cross-linkers and cross-linked ligands are replaced by the added ligand mixture (PbBr_2_/OA/OLAm). The postpatterning surface treatment is also applicable to other types of perovskite NCs and successful in the recovery of PLQYs for DOPPLCER-patterned FAPbBr_3_ NCs (Fig. 5, C and D) and CsPbBr_3_ NCs with zwitterionic ligands (fig. S20). The above results suggest that the compelling set of features of DOPPLCER, especially the mild condition, native ligand chemistry, and compatibility with postpatterning surface treatment, allows for the preservation of the optical properties of pristine perovskite NCs and thus promises the fabrication of high-performance optoelectronic devices.

**Fig. 6.**
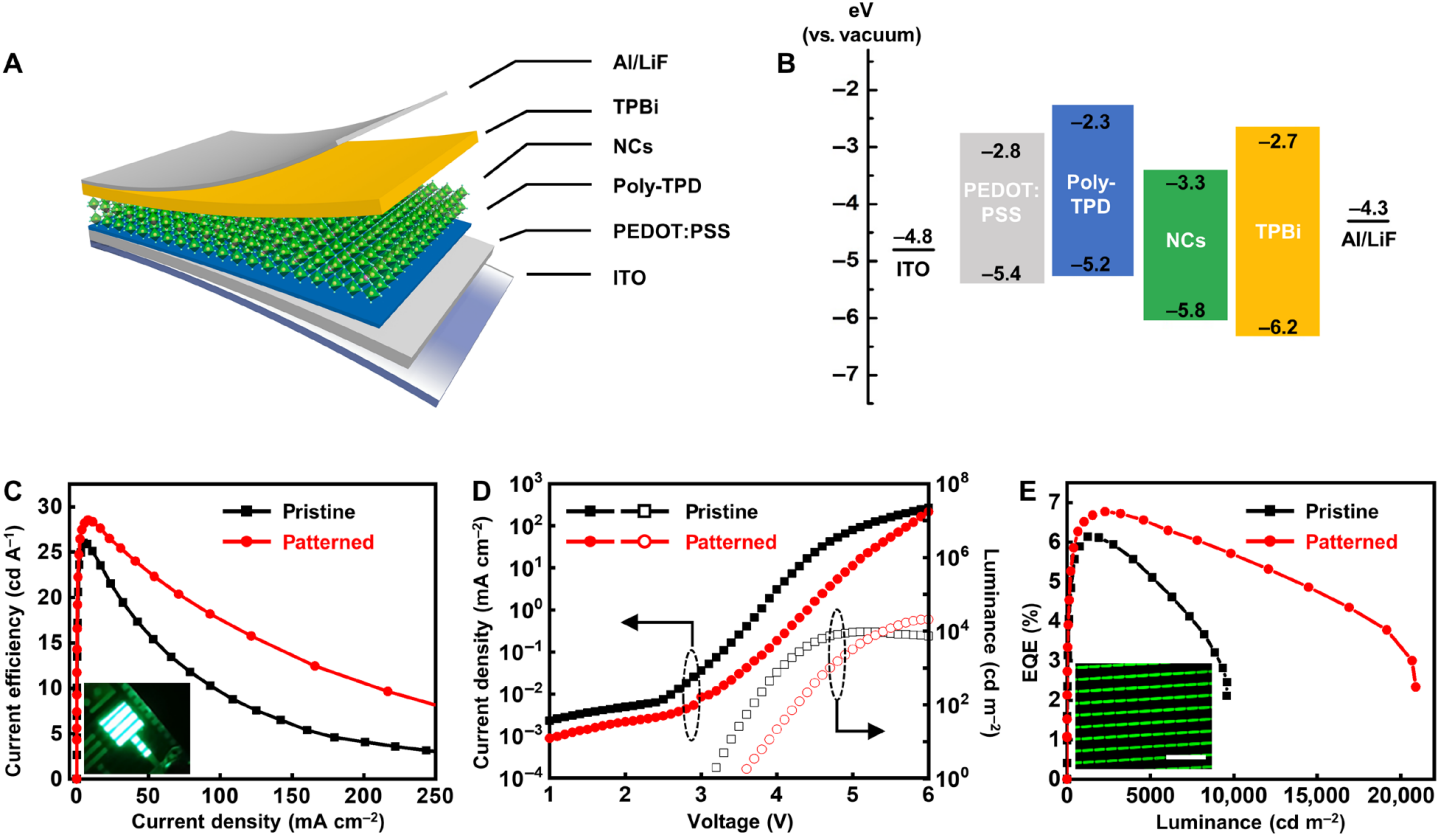
EL characteristics of FAPbBr_3_ NC LEDs processed by DOPPLCER. (**A** and **B**) Schematic device structure and energy band diagram of a representative LED. (**C**) Current efficiency–current density, (**D**) Current density–voltage–luminance, and (**E**) EQE characteristics of pristine and patterned devices. Insets in (C) and (E) show the photo of a patterned, working device and an EL image of a pixelated NC LED. Scale bar, 200 μm (E). Photo credit: Dan Liu and Fu Li, Tsinghua University.

The steps of DOPPLCER adapt well to the established workflows for fabricating EL devices. To show the potential for patterning display pixels, we fabricated prototype EL devices with CsPbBr_3_ and FAPbBr_3_ NCs as the active layers. A typical device structure includes stacked layers of indium tin oxide cathode (ITO), poly(3,4-ethylenedioxythiophene) polystyrene sulfonate (PEDOT:PSS; 25 nm), poly[bis(4-phenyl)(4-butylphenyl)amine] (poly-TPD) (8 nm), NCs, 2,2’,2”-(1,3,5-Benzinetriyl)-tris(1-phenyl-1-H-benzimidazole) (TPBi) (35 nm), and LiF (1 nm)/Al (Fig. 6A) (*44*). Figure 6B shows the schematic energy level diagram. The active layer was made by either (i) spin-coating the pristine NC ink (pristine devices) or (ii) spin-coating NC ink containing bisFPA molecules, followed by UV exposure and developing steps (patterned devices). For the latter type of devices (ii), the entire active layer was exposed for the simplicity in comparing device performance. The exposure dose was 60 mJ cm^−2^, while the ratio of bisFPA to NCs was 10 and 20 wt% for FAPbBr_3_ and CsPbBr_3_ NCs, respectively. All device fabrication and DOPPLCER-related procedures were performed under inert atmosphere in a nitrogen-filled glove box. Figure 6 (C to E) and table S5 compare the EL device parameters of pristine and patterned devices with FAPbBr_3_ NCs. The EL emission peak of patterned devices remains centered at ~527 nm (full width at half maximum, 24 nm) without noticeable broadening or shift in peak position (fig. S21). Patterned FAPbBr_3_ NC LEDs compare favorably over the pristine ones, with similar current efficiency (28.6 versus 26.0 cd A^−1^; Fig. 6C) and more than twofold increase in maximum luminance (2 × 10^4^ versus 9500 cd m^−2^; Fig. 6D). The EQEs of patterned devices reach a maximum of 6.8% at a brightness of 2350 cd m^−2^ and compare favorably with those of pristine devices over a wide range of brightness/current densities (Fig. 6E). Note that the patterned devices contain an active layer made from two consecutive coating and patterning of FAPbBr_3_ NCs for improved performance. Patterned devices with a single coated layer of FAPbBr_3_ NCs (fig. S22 and table S5) show an EQE of 5.7% and a maximum luminance of 4700 cd m^−2^ that are on par with those of pristine devices. The peak EQE and maximum luminance of the prototype patterned devices are among the highest of reported values for patterned perovskite LEDs of NCs, single crystals, and polycrystalline films, made by solution-based process or vacuum deposition (*49*). Details of these reported EL characteristics and corresponding patterning methods are summarized in table S6. The EL characteristics of prototype pristine and patterned devices are lower than the record values (e.g., EQEs over 20%). Preliminary tests on device operation stability show that the luminance drops to 50% of its initial value (initial luminance, ~100 cd m^−2^) in 5 to 7 min for both pristine and patterned devices, in the same order as those reported for FAPbBr_3_ NC LEDs (*44*). Considering the rapid increase in reported EQEs for perovskite NC–based LEDs, it is conceivable that the performance of both pristine and DOPPLCER-patterned devices can be remarkably improved after optimization. Some potential strategies are listed in Discussion. To demonstrate the generality of DOPPLCER in building perovskite NC LEDs, we also compared the characteristics of pristine and patterned devices with CsPbBr_3_ NCs as the active layer. As shown in fig. S23 and table S5, these two types of devices display almost identical EL performance. The highest brightness for patterned devices is 1929 cd m^−2^, and the maximum EQE is 1.8%. Despite the variation in device characteristics due to the use of different perovskite NCs, these results suggest that DOPPLCER does not degrade the EL device performance. Last, we successfully made proof-of-concept, pixelated LEDs (inset in Fig. 6E). The dimension of each pixel is 10 μm by 50 μm. This shows the potential of DOPPLCER in realizing EL displays.

In addition, DOPPLCER is also potentially applicable to build perovskite image sensors consisting of an array of NC-based photodetectors. As a proof of concept, we fabricated pristine and patterned CsPbBr_3_ NC thin-film photoconductors with simple device structures, following procedures similar to those used in constructing active layers of LEDs. Compared to LEDs, the performance of photoconductors relies critically on the interparticle electronic coupling and charge transport behavior, which are typically hindered by the native, aliphatic ligands of NCs. As a consequence, both the pristine and patterned thin films of CsPbBr_3_ NCs show small photocurrents at nanoampere level. A set of solid-state ligand exchange processes, proposed by Wheeler and co-workers (*50*) for making photovoltaics with record efficiency, can replace the native ligands for shorter ions/species, leading to about two orders of magnitude increase in photocurrents (to about 4 μA when illuminated with 450-nm light at 13.0 mW cm^−2^) for NC films (fig. S24). Patterned NC films after these postpatterning treatments also show photocurrents comparable to those of pristine devices (300 versus 400 nA, at 0.8 mW cm^−2^ illumination). This suggests that the native aliphatic ligands, even after being photocrosslinked via intermolecular covalent C─N bonds, can be easily replaced by compact ligands from the NC surface. FTIR spectra in fig. S24 confirmed the ligand exchange process, as evidenced by the suppressed vibrational modes for C─H (~2800 to 3000 cm^−1^) and the occurrence of C═N (1715 cm^−1^) and C═N─H (3350 cm^−1^) modes related to formamidinium cations. The compatibility with postpatterning ligand exchange strategies promises the use of DOPPLCER in electronic and photonic devices reliant on facile inter-NC charge transport. Future work is needed to evaluate the conductivities, carrier mobilities, and other parameters important for inter-NC electronic communication.

## DISCUSSION

The DOPPLCER method presented here enables straightforward, fast, scalable, and high-fidelity patterning of perovskite NCs with their native ligands. The nonspecific nature of the underlying nitrene photocrosslinking chemistry translates to a broad applicability to inorganic and hybrid perovskite NCs with various core properties and surface chemistry, as well as the capability in multicolored patterning in a layer-by-layer fashion. The freedom in the choice of ligands distinguishes DOPPLCER from other direct patterning methods reliant on judicious selection of photoactive ligands. The mild condition follows from the highly efficient photocrosslinking chemistry and yields CsPbBr_3_ and FAPbBr_3_ NC patterns with moderate decrease in PLQY, which can be fully recovered after postpatterning ligand exchange (absolute PLQY, ~76%). DOPPLCER can be seamlessly integrated with the workflows for fabricating pixelated devices that are prerequisites for the practical, system-level applications. Prototype LEDs with patterned NC layers show a peak EQE of 6.8% and a maximum brightness over 2 × 10^4^ cd m^−2^, both are among the highest values reported for patterned perovskite LEDs. Preliminary photoconductor devices also show decent photoresponse thanks to the compatibility of DOPPLCER with established surface treatment strategies.

DOPPLCER represents a powerful approach compared to other patterning methods for perovskite NCs (table S7). Further optimizations on the cross-linking chemistry and device parameters are expected to achieve more quantitative retention of PLQY and substantially improved device performance. From the ligand chemistry perspective, chemical design of the bisFPA molecules and other types of photocrosslinkers would allow patterning at longer wavelengths (and potentially less photodamage), with a smaller amount of cross-linkers or lower doses and with higher efficiencies. These may reduce the generation of surface traps and other detrimental effects during patterning and realize nondestructive patterning of perovskite NCs. For instance, our preliminary experiment shows that CsPbBr_3_ NC films fully retain their PLQYs after being exposed to 365 nm for 60 mJ cm^−2^. Figure S25 shows an example of these designed photocrosslinkers that enable NC patterning at 365 nm. Photogenerated carbene and related chemistry, recently adopted to cross-link inert polymers and pattern polymeric organic electronic circuits (*51*), can work as well for patterning perovskite NCs. The subtle differences in the underlying photochemistry and reaction kinetics of photogenerated nitrene and carbene may offer an additional degree of tunability in DOPPLCER. The development of new cross-linkers is a subject of on-going work. From the device aspect, impressive progress in the performance of perovskite NC–based optoelectronic devices has been made during the past years thanks to the tremendous efforts in the optimization of NC cores, surface states, and device structures. Among them, surface engineering with desirable ligands has been proven effective to achieve high-efficiency and stable perovskite NC LEDs (*44*, *52*). The broad applicability of DOPPLCER promises its potential in constructing highly pixelated LEDs and independently addressable photodetector arrays with device characteristics approaching the reported records (e.g., EQE ~ 20% for LEDs). We expect DOPPLCER to open a new avenue for bridging the recent advancements on perovskite NCs at material and single device level and the need for the practical applications in diverse, system-level electronic and optoelectronic platforms, such as high-resolution displays, image sensors, and wearable devices.

## MATERIALS AND METHODS

### Perovskite NC synthesis

CsPbBr_3_ NCs capped with OLAm and OA were synthesized using procedures similar to previous reports (*36*). Briefly, 200 mg of PbBr_2_, 3 ml of OLAm, 1.5 ml of OA, and 15 ml of ODE were loaded in a 50-ml three-necked flask and dried under vacuum at 120°C for 1 hour. After complete dissolution of PbBr_2_, the solution was heated to 170°C under nitrogen. Once the temperature was stable, 1.2 ml of cesium oleate (the preparation method shown in the Supplementary Materials) was swiftly injected. After 5 s, the reaction mixture was cooled by ice-water bath, followed by the purification procedures described below. CsPbBr_3_ NCs with an average size of ~3.9 nm and blue emission were synthesized by using a method described by Dong *et al.* (*7*). PbBr_2_ (175 mg), 350 mg of ZnBr_2_, 4 ml of OA, 5 ml of OLAm, and 10 ml of ODE were loaded in a 50-ml three-necked flask and degassed for 1 hour at 120°C. The reaction mixture was cooled to 80°C, followed by the rapid injection of 0.8 ml of cesium oleate. The reaction was stopped after 3 min and cooled with an ice water bath. Organic-inorganic hybrid FAPbBr_3_ NCs were synthesized by a LARP method reported elsewhere (*44*). A precursor solution containing 0.027 g of FABr, 0.0367 g of PbBr_2_, 250 μl of OA, 25 μl of octylamine, and 500 μl of dimethylformamide was loaded in a 2-ml glass vial and then heated at 50°C under stirring until PbBr_2_ was completely solubilized. The precursor solution was then swiftly injected to a 20-ml glass vial with 8 ml of chloroform under vigorous stirring. After 35 s, the reaction was quenched by adding 2 ml of acetonitrile. The resultant solution was centrifuged at 10,000 rpm for 5 min, and FAPbBr_3_ NC precipitates were collected. The NCs were redispersed in 2 ml of toluene and centrifuged at 4000 rpm for 2 min to remove insoluble impurities. The supernatant was collected and stored in the dark at 4°C for further use. The synthesis of CsPbBr_3_ NCs capped with OPA (*38*) and zwitterion ligands (*42*) and CsPbCl_3−*x*_Br*_x_* and CsPbI_3−*x*_Br*_x_* NCs with mixed anions were described in the Supplementary Materials. After purification, all perovskite NCs were dispersed in anhydrous toluene or hexane and stored under inert atmosphere.

### Synthesis of bisFPA photocrosslinkers

Ethylene bis(4-azido-2,3,5,6-tetrafluorobenzoate) with optical absorption maximum at around 260 nm was synthesized according to the recipe reported by Cai *et al.* (*32*). Detailed synthetic methods and characterizations of these molecules appear in the Supplementary Materials. These bisFPA molecules were kept at −10°C in the dark in a glove box. Caution should be taken, as sodium azide used in the synthesis is toxic and explosive when shocked or heated at high temperatures. It must be handled with care.

### Purification procedures for CsPbBr_3_ NCs with OLAm and OA ligands

After synthesis, NCs were isolated from the crude solution by adding methyl acetate (MeOAc; crude solution: MeOAc = 1:3, v/v), followed by centrifugation at 10,000 rpm for 5 min. The supernatant was discarded, while the NC pellets were redispersed in 15 ml of anhydrous hexane for further purification steps. The subsequent purification procedures were performed in the nitrogen-filled glove box with anhydrous hexane (or toluene) and MeOAc as the pair of solvent/nonsolvent. Purification procedures denoted as A, B, and C used one cycle of purification with different amounts of MeOAc [MeOAc:hexane = 1:1, 2:1, and 3:1 (v/v) for procedures A, B, and C, respectively]. Purification procedures denoted as D, E, and F used involved an additional cycle of purification (MeOAc:hexane = 1:1 in volume) compared to procedures A, B, and C, respectively.

### Procedures for DOPPLCER

All procedures for DOPPLCER were performed under yellow light typically used for clean room lighting.

1) Spin coating of perovskite NCs. In a typical DOPPLCER procedure, CsPbBr_3_ NCs synthesized with OLAm and OA as the ligands and purified with procedures described above were dispersed in toluene (20 mg ml^−1^). bisFPA solution (10 mg ml^−1^) was prepared by dissolving bisFPA powders in toluene. A desirable amount of bisFPA (typically 10 to 20 wt% of the mass of NCs) was added to NC solution shortly before spin coating. The formed NC ink was spin-coated on cleaned substrates (silicon, quartz, glass, or flexible polyimide) at 2000 rpm for 30 s. This protocol yielded films with thickness of about 30 nm. The thickness of spin-coated thin films can be tuned by changing the concentration of NCs and/or spin coating parameters.

2) UV exposure. The coated thin films were irradiated with UV lamps with peak intensities at 254 (3 mW cm^−2^) or 365 nm (3 mW cm^−2^), depending on the choice of bisFPA cross-linkers. The exposure dose at 254 nm was typically 60 mJ cm^−2^. Patterned NC thin films were obtained by irradiating thin films through a quartz mask with predesigned patterns in a mask aligner system or using binder clips [as described by Wang *et al.* (*26*)].

3) Developing. These thin films were then developed by nonpolar solvents (toluene, hexane, or octane) to remove/redisperse NCs in the unexposed regions. This step can be performed either by immersing thin films in the developer solvents (typically within 3 min) or by dynamic spin coating. For the latter, the UV-exposed films were fixed on the spinner chuck by vacuum, followed by spinning at 2000 rpm for 10 s. During this process, 100 μl of toluene or hexane was added dropwise to the films. The patterning of FAPbBr_3_ NCs followed similar procedures except for the changes in the mass ratio of bisFPA (10 wt%) and spin coating parameters (1000 rpm, 50 s). Parameters for DOPPLCER varied for perovskite NCs with different sizes, compositions, and surface ligands.

### Fabrication and characterization of pristine and patterned LEDs

Pristine and patterned FAPbBr_3_ and CsPbBr_3_ NC-based LED devices were fabricated in a structure of ITO/PEDOT:PSS (25 nm)/poly-TPD (8 nm)/active layer (pristine or patterned)/TPBi (35 nm)/LiF (1 nm)/Al. The ITO substrates were cleaned by a sequence of ultrasonication with detergent, distilled water, and ethanol and then stored in ethanol solution. Before device fabrication, the ITO substrates were dried under nitrogen flow and subjected to UV-ozone treatments for 30 min. PEDOT:PSS solution filtered by a 0.45-μm PTFE filter was then spin-coated onto ITO substrates at 4000 rpm for 30 s and baked at 150°C for 15 min. The hole-transporting layer was spin-coated by using a solution of poly-TPD in chlorobenzene (7 mg ml^−1^) at 3500 rpm for 30 s. Optionally, a small amount of bisFPA can be added to the solution of poly-TPD (1 wt%) to reduce the redissolution of poly-TPD during the subsequent coating and patterning process of NCs, followed by UV irradiation (254 nm, 3 mW cm^−2^ for 100 s) and post-exposure bake at 80°C for 10 min. For FAPbBr_3_ NC-based LEDs, NC solution in toluene (15 mg ml^−1^) with 10 wt% of bisFPA additives was spin-coated at 2000 rpm for 30 s. For patterned devices, the NC layer was exposed with a dose of 60 mJ cm^−2^ (254 nm, 3 mW cm^−2^, 20 s) with or without a predesigned photomask. Afterward, the films were developed by spin-coating octane to remove unreacted bisFPA and unexposed NCs, by using the dynamic spin-coating process described above, followed by post-bake at 70°C for 5 min. The NC coating/exposure/developing process can be repeated to make patterned devices with two consecutive layers of FAPbBr_3_ in the active layer. TPBi and LiF/Al electrodes were thermally evaporated through a shadow mask with the device area of 7.25 mm^2^. The preparation of CsPbBr_3_ NC-based devices followed similar procedures but typically with a larger amount of bisFPA (20 wt%). All devices were fabricated and measured in nitrogen. The EL characteristics and spectra were measured with a Keithley 2400 source meter in conjunction with an absolute EQE measurement system (C992012, Hamamatsu Photonics K.K.).

### Fabrication and characterization of pristine and patterned photoconductors

The substrates with interdigitated Au electrodes (channel width, 10 μm; total channel length, 0.49 mm; and thickness of electrodes, 50 nm) were fabricated on silicon substrate by standard photolithography and evaporation processes. CsPbBr_3_ NCs (60 μl; 20 mg ml^−1^) were spin-coated on the cleaned substrates at 800 rpm for 60 s. Compared to pristine devices, the patterned devices used NC solutions with bisFPA (10 wt%), followed by UV irradiation (254 nm, 1.5 mW cm^−2^, 30 s), and developed by rinsing with *n*-hexane and an additional rinse with 1 ml of MeOAc. The entire film was exposed for patterned devices for simplicity in evaluating device performance. Ligand exchange process was performed for both types of devices following a modified protocol developed by Wheeler and co-workers (*50*). Briefly, the films were soaked in a saturated Pb(NO_3_)_2_ solution in MeOAc for 30 s. Excess Pb(NO_3_)_2_ was removed by rinsing with 1 ml of MeOAc. Both types of devices comprised three spin-coated layers of NCs with ligand exchange and patterning procedures performed between two consecutive coatings. The obtained three-layered devices were then soaked in a saturated FABr solution in ethyl acetate for 90 s, followed by rinsing with 1 ml of MeOAc to remove excess FABr. The current-voltage characteristics of these devices were collected by a semiconductor analyzer (Agilent B1500) in the dark or under illumination (450 nm with tunable light intensity). The devices were fabricated in a nitrogen-filled glove box and measured under ambient condition.

### Characterization techniques

Details in characterization methods are shown in the Supplementary Materials.

## Supplementary Materials

### This PDF file includes:

Supplementary Text Figs. S1 to S25 Tables S1 to S7 References
