# Supplementary material for: Direct optical patterning of perovskite nanocrystals with ligand cross-linkers
Source: Sci Adv. 2022 Mar 16;8(11):eabm8433. doi: 10.1126/sciadv.abm8433 (PMC8926341; doi:10.1126/sciadv.abm8433)
Supplement: Supplementary file 1 — Supplementary Text Figs. S1 to S25 Tables S1 to S7 References [file sciadv.abm8433_sm.pdf]

Science Advances
AAAS

# Supplementary Materials for

## Direct optical patterning of perovskite nanocrystals with ligand cross-linkers

Dan Liu, Kangkang Weng, Shaoyong Lu, Fu Li, Hannikezi Abudukeremu,
Lipeng Zhang, Yuchen Yang, Junyang Hou, Hengwei Qiu, Zhong Fu, Xiyu Luo, Lian Duan,
Youyu Zhang*, Hao Zhang*, Jinghong Li

*Corresponding author. Email: zhangyy@hunnu.edu.cn (Y.Z.); hzhangchem@mail.tsinghua.edu.cn (H.Z.)

Published 16 March 2022, *Sci. Adv.* **8**, eabm8433 (2022)
DOI: 10.1126/sciadv.abm8433

**This PDF file includes:**

Supplementary Text
Figs. S1 to S25
Tables S1 to S7
References

**Supplementary Text**

**Supplementary Methods**

**1. Chemicals.**

**Nanocrystal synthesis, ligand exchange and anion exchange.** Cesium carbonate ($Cs_2CO_3$, 99.9%, trace metals basis), lead acetate trihydrate ($Pb(CH_3COO)_2$ $3H_2O$, 99.999%, trace metals basis), trimethylsilyl bromide (TMSBr, 97%), octylamine (OTAm, 99%), trioctylphosphine oxide (TOPO, 90%), methyl acetate (MeOAc, anhydrous, 99.5%), ethyl acetate (EtOAc, anhydrous, 99.5%) and hexane (anhydrous, 95%) were purchased from Sigma-Aldrich. Lead (II) bromide ($PbBr_2$, 99.999%, metals basis), zinc bromide ($ZnBr_2$, anhydrous, 99.99%, metals basis), N,N-Dimethylformamide (DMF, 99.8%), octylphosphonic acid (OPA, 98%), oleic acid (OA, tech. 90%), and octadecene (ODE, tech. 90%) were from Alfa Aesar. Benzoyl bromide (98%), benzoyl chloride (98%, GC&T), Formamidine bromide (FABr, 99.99%), lead (II) iodine ($PbI_2$, 99.999%, metals basis), and octadecyldimethyl(3-sulfopropyl)ammonium hydroxide inner salt (DMOA-PS, >97%) were from TCI. Oleylamine (OLAm, C18: 80-90%) was from Acros. Other chemicals and solvents involved in the synthesis and anion exchange of NCs are chloroform ($CH_3Cl_3$, 90%, Innochem), acetonitrile (99.9%, Fisher), cesium acetate (99%, innochem), potassium acetate ($CH_3COOK$, 99.0%, AR, Macklin), trimethylsilyl iodide (TMSI, 97%, with Cu as a stabilizer, Aladdin). Anhydrous toluene was obtained by using a solvent purification system. OA, OLAm, and ODE were degassed prior to use in the glove box. All other chemicals were used as received.

**Synthesis of bisFPAs.** Methyl 2,3,4,5,6-pentafluorobenzoate (97%, Heowns), sodium azide ($NaN_3$, J&K), 4-(dimethylamino) pyridine (DMAP, 98%, Energy Chemical), N′-ethyl-N-(3-(dimethylamino)propyl) carbodiimide hydrochloride (EDC, 99%, J&K), magnesium sulfate (98%, Innochem), sodium hydroxide (99%, Innochem), hydrochloric acid (37% solution in water, Tongguang Fine Chemicals), ethylene glycol (99%, Innochem), chloroform (90%, Innochem), dichloromethane ((AR, Innochem), acetone (AR grade, General Reagent), diethyl ether (AR grade,

General Reagent), methanol (AR grade, General Reagent), deionized water (General Reagent), silica gel (100–200 mesh, Innochem). All chemicals were used as received.

**Chemicals for LED Devices.** PEDOT:PSS solution (CLEVIOSTM PVP AI4083, Heraeus), poly[bis(4-phenyl)(4-butylphenyl)amine] (poly-TPD, Xi'an Polymer Light Technology), 2,2',2"-(1,3,5-Benzinetriyl)-tris(1-phenyl-1-H-benzimidazole) (TPBi, Jilin Optical and Electronic Materials), lithium fluoride (LiF, 99%, Alfa Aesar), Aluminum (Al, Sinopharm Chemical Reagent). All reagents were used without further purification.

**2. NC synthesis.**

**Preparation of cesium oleate (0.125 M) solution** (*36*). 0.814 g of $Cs_2CO_3$, 2.5 mL of OA and 40 mL of ODE were loaded in a 100 mL 3-necked flask and degassed at 120 $^oC$ for 1 h. Under nitrogen, the flask was then heated to 150 $^oC$ until complete reaction of $Cs_2CO_3$ with OA, as shown by the formation of a homogeneous solution. The cesium-oleate solution was transferred to a nitrogen-filled glovebox and stored under inert atmosphere. The stock solution needed to be heated to 100 ℃ to form a homogeneous solution before use.

**$CsPbBr_3$ NCs with other ligands.** NCs with zwitterionic ligands (*42*) were synthesized following a reported method. First, cesium oleate (2.4 mL, 0.4 M), lead oleate (3 mL, 0.5 M), DMOA-PS (0.6 mmol) and ODE (30 mL) were mixed in a 100 mL 3-neck flask under Schlenk lines and then degassed at 120 ℃ for 1 hour. Under nitrogen, the flask was heated to 170 ℃ and TMSBr (2.9 mmol) was injected. The heating mantle was immediately removed and the reaction was quenched by cooling with an ice-water bath. The crude solution was purified by addition of EtOAc (solution: EtOAc = 1:1 in volume) and centrifuged at 8500 rpm for 5 min. The NC precipitates were redispersed in toluene. The purification step was repeated once. Purified NCs were dispersed in toluene for the following patterning processes. NCs with OPA ligands were synthesized according to another report (*38*). First, 0.1 g of OPA, 1 g of TOPO, 0.069 g of $PbBr_2$ and 5 mL of ODE were loaded in a 50 mL 3-neck flask and then degassed under vacuum at 100 $^oC$

for 1 h. Then the solution was heated to 170 °C under nitrogen, followed by swift injection of 0.4 mL of cesium oleate. The reaction lasted for about 5 s and was then quenched by using an ice-water bath. For purification, an equal volume of MeOAc was added to the crude solution and the NCs were separated by centrifugation at 8500 rpm for 5 min. NCs were redispersed in toluene for further use.

**$CsPbCl_{3-x}Br_3$ NCs (*53*).** 32 mg of $Cs_2CO_3$, 152 mg of $Pb(CH_3COO)_2\cdot 3H_2O$, 0.6 mL of OA, 2 mL of OLAm and 10 mL of ODE were loaded in a 50 mL 3-necked flask and dried under vacuum for 1 h at 120 °C. Subsequently, the temperature was raised to 180 °C under nitrogen. At this temperature, benzoly halide precursors were swiftly injected (100 μL of benzoyl bromide and 100 μL of benzoyl chloride). The reaction was stopped after 5 s by removing the heating sources and quenching with an ice-water bath. The crude solution was transferred to a nitrogen-filled glovebox and purified twice by using hexane/MeOAc as the solvent/nonsolvent pair.

**$CsPbI_{3-x}Br_3$ NCs (*54*).** A precusor solution of potassium oleate was prepared by heating a mixture of $CH_3COOK$ (0.1 mmol), OA (0.5 mL) and ODE (4.5 mL) at 80 °C for 30 min. After cooled to room temperature, the solution was kept in a nitrogen-filled glove box. 0.2 mmol of $Pb(CH_3COO)_2\cdot 3H_2O$, 0.1 mmol of $CH_3COOCs$, 0.5 mL of OA, 1 mL of OLAm, 1 mL of potassium oleate solution and 4.0 mL of ODE were mixed in a 50 mL 3-neck flask and degassed at 120 °C for 1 h,. Then the temperature was raised to 180 °C under nitrogen. Halide precursors were swiftly injected (110 μL of TMSI and 58 μL of TMSBr). After 10 s, the flask was cooled with ice water. The purified procedure was the same to that of $CsPbCl_{3-x}Br_3$ NCs.

**3. Synthesis of bisFPAs.** Ethylene bis(4-azido-2,3,5,6-tetrafluorobenzoate) was synthesized according to reported protocols (*32*). In a flask, 40 mL of acetone and 15 mL of water were added to a mixture of $NaN_3$ (23 mmol, 1.50 g) and methyl 2,3,4,5,6-pentafluorobenzoate (**1**) (21.5 mmol, 4.86 g) under stirring. The formed solution was then refluxed for 8 h and cooled to room temperature. 30 mL of water was added to the solution, followed by extraction with diethyl

ether three times (3 × 50 mL). The extract was dried over anhydrous $MgSO_4$. After solvent removal by rotary evaporation, methyl 4-azido-2,3,5,6-tetrafluorobenzoate (**2**) (5.23 g, yield ~97%) was obtained as a red liquid.

A solution of 4.99 g of **2** in methanol (85 mL) was added with NaOH (7 mL, 20% aqueous solution) and stirred overnight at 25 $^oC$. The solution was acidified by 2N HCl in an ice bath until pH < 1, followed by extraction by $CHCl_3$ (3 × 50 mL). The extract was dried over anhydrous $MgSO_4$ and evaporated under reduced pressure. 4.29 g (yield ~86%) of methyl 4-azido-2,3,5,6-tetrafluorobenzoic acid (**3**) was obtained as a colorless solid.

A solution of (**3**) (2.50 g, 10.75 mmol) in dry $CH_2Cl_2$ (50 mL) was stirred with ethylene glycol (0.33 g, 5.25 mmol) and DMAP (0.13 g, 1.08 mmol) under nitrogen at room temperature for 30 min. Afterward, EDC (2.27 g, 14.6 mmol) was added to the solution. The mixture was stirred overnight at room temperature. Then 30 mL of water was added. After stirring for 30 min at room temperature, the mixture was extracted with 50 mL of $CH_2Cl_2$ for 3 times. The combined organic layers were washed with water (3 × 100 mL), brine (100 mL) and dried over $MgSO_4$. The compound was purified by column chromatography (silica gel, eluting solvent 3:2 hexane:ethyl acetate) to give a white solid of bisFPA (1.11 g, yield ~50%). $^1H$ NMR (400 MHz, $CDCl_3$): $\delta \sim 4.68$ (s, 4H); $^{19}F$ NMR (377 MHz, $CDCl_3$): $\delta \sim -150.81$ to $-150.70$, (m, 4F) and $-138.27$ to $-138.18$ (m, 4F).

Synthesis of bisFPA that are sensitive at 365 nm used a reported protocol (*55*).

**4. Characterization techniques.** Transmission electron microscopy (TEM) images of NCs were obtained using a JEOL JEM-2100F microscope. Scanning electron microscopy (SEM) measurements of pristine and patterned films were carried out on a Hitachi SU-08010 microscope at 10 kV. The atomic force microscopy (AFM) images were taken on Oxford Cypher 5. Optical microscopic images of NC patterns in bright and fluorescent modes were taken with a Nikon Ni-U microscope. The UV-Vis absorption spectra of NC solutions and thin films were collected using a Cary 5000 UV-Vis-NIR spectrophotometer in the transmission mode. The PL emission spectra of

solution and film samples were obtained by a Horiba FluoroMax Plus spectrometer. Fourier-transform infrared (FTIR) spectra of films composed of NCs and bisFPA were collected using a Bruker VERTEX 70 spectrometer in a transmission mode. The samples for FTIR measurements were prepared on by spin-coating NC solutions (with or without bisFPA) in toluene on a KBr crystal substrate and exposed to different UV doses to monitor the photolysis of bisFPAs. X-ray diffraction (XRD) of NC thin film were collected by using a Rigaku Smartlab diffractometer with Cu $K_\alpha$ radiation. X-ray photoelectron spectroscopy (XPS) and ultraviolet photoemission spectroscopy (UPS) measurements were performed on a ThermoFisher Scientific ESCALAB $Xi^+$ spectrometer using a monochromatic Al $K_\alpha$ and He I (21.2 eV) sources, respectively. The samples were prepared by spin-coating the corresponding solution on a gold-coated (50 nm) silicon wafer. The HOMO energy was obtained directly from the UPS results and calculated by the following equation, $E_{HOMO} = h\upsilon - (E_{cutoff} - E_{onset})$. $^1$H-NMR spectra of NCs, $^1$H- and $^{19}$F-NMR spectra of bisFPA were collected by a JEOL ECS-400 at 400 MHz. $CsPbBr_3$ NCs capped with OLAm/OA and purified with different protocols were dispersed in hexane. The solutions were dried thoroughly, re-dispersed in 700 μL of $CDCl_3$ and transferred into NMR tubes. Ferrocene with a known concentration ($3.2 \times 10^{-4}$ mM) was added as an internal standard. The NMR spectra were taken with relaxation time of 20 s and scan times of 32. The height profiles of line patterns of NCs were estimated by a Bruker Dektak-XT instrument. Inductively coupled plasma optical emission spectroscopy (ICP-OES) analysis was carried out on a Varian Vista-MPX spectrometer. To estimate the film retention of patterned films at different conditions, NCs remained in the films and recovered from developer solvents were digested in an acidic condition and diluted with deionized water. Absolute PLQYs of pristine and DOPPLCER treated NC films (on quartz substrates) were monitored by Horiba FluoroMax Plus spectrometer with a 3.2 inch-integrating sphere. The emission spectrum of a quartz substrate was collected as the blank. The excitation wavelength was set to 365 nm and the emission spectra ranges from 330 to 610 nm. The measurements provided the intensities

of fluorescence emission ($E_{sample}$ and $E_{\mathrm{blank}}$) at the emission peak wavelength and scattered light ($L_{sample}$ and $L_{\mathrm{blank}}$) at the excitation wavelength. The absolute PLQYs can be calculated from the following equation, $\Phi_f = \frac{E_{sample} - E_{blank}}{L_{blank} - L_{sample}} \times 100\%$. Time-resolved PL decay signals were recorded by using a time-correlated single photon counting (TCSPC) method. Data was collected on a Horiba FluoroMax Plus spectrometer equipped with a DeltaHub TCSPC-MCS kit. NC thin films were excited by a pulsed LED (350 ±10 nm) and the emission intensities at their maximum emission wavelengths were monitored.

**Extended description of the results**

**Comparison of the absorption coefficients of $CsPbBr_3$ NCs and bisFPA.** According to the work by Hens and co-workers (*41*), the intrinsic absorption coefficient of $CsPbBr_3$ NCs at 335 nm ($\mu_{i,335}$ = 1.59 × $10^{-5}$ $cm^{-1}$) is independent of the NC sizes. The molar absorption coefficient at 335 nm ($\varepsilon_{335}$) for NCs with an average size of 7.7 nm is 1.9 × $10^7$ $cm^{-1}$ $M^{-1}$, calculated by $\varepsilon_{NC,335} = \frac{N_A V_{NC} \mu_{i,335}}{\ln 10}$, where $N_A$ is the Avogadro number, $V_{NC}$ is the volume of a single NC (approximately the cubic of the size of NCs). Based on the absorption spectrum of NCs (Fig. 2A), the molar absorption coefficient of NCs at 264 nm ($\varepsilon_{NC,264}$) can be extrapolated (5.2 × $10^7$ $cm^{-1}$ $M^{-1}$). Although $\varepsilon_{NC,264}$ is about 3 orders of magnitude higher than that of bisFPA ($\varepsilon_{264}$ of bisFPA ~2.9 × $10^4$ $cm^{-1}$ $M^{-1}$), $\varepsilon_{264}$ for a unit of [$CsPbBr_3$] in the NCs is about the same (~2.3 × $10^4$ $cm^{-1}$ $M^{-1}$). This was estimated by using $\varepsilon_{[CsPbBr3]} = \frac{\varepsilon_{NC}}{number\ of\ [CsPbBr3]\ in\ a\ single\ NC} = \frac{\varepsilon_{NC}}{(d^3/a^3)}$, where $d$ is the average size of NCs and $a$ is the size of a unit cell ($a$ = 0.587 nm). In a mixed solution containing $CsPbBr_3$ NCs and bisFPA (the mass ratio of bisFPA to NCs is 20 wt.%), the absorption from bisFPA is estimated to be about 28% of that from NCs. This ratio is close to the experimental results shown in Fig. 2A (~20%). The above analysis suggested that the high absorption coefficient of bisFPA molecules ensures their fast photolysis and generation of nitrene radicals in the NC thin films, despite the large absorption efficient of perovskite NCs.

**Calculation of the ligand densities and ratios between ligands and bisFPA on NCs with different purification procedures.** In Table S1, Purification procedures A to F were described in the Materials and Methods. The molar concentration of oleyl species accounts for the sum of OLAm and OA. This was estimated by the integral of alkene resonances in NMR measurements with ferrocene ($3.2 \times 10^{-4}$ mM) as an internal standard. The molar ratio between ODE and oleyl species was obtained by comparing the integral of their alkene resonances in NMR spectra. The optical absorbance at 335 nm of $CsPbBr_3$ NCs was measured for diluted NC solutions (60 ×), which allowed for the calculation of NC concentration by using the reported formula (*37, 41*). The concentration of NCs, $[\mathrm{NC}] = \frac{f}{d^3 N_A} = \frac{A \times \ln 10}{(\mu_i \times L)(d^3 N_A)}$, where $f$ is the volume fraction of the NC material, $d$ is the size of NCs ($d$ = 7.7 nm), $N_A$ is the Avogadro number, $\mu_i$ is the intrinsic absorption coefficient of NCs ($\mu_i = 1.59 \times 10^5$ cm$^{-1}$ at 335 nm, independent of the NC sizes), $L$ is the optical length (1 cm). The nominal ligand density was calculated based on the concentration of NCs and ligands, by dividing the number of oleyl molecules per NC by the surface area of NCs (defined as $6d^2$). The number of bisFPA per NC was calculated by the following formula,

$$\frac{number\ of\ bisFPA}{number\ of\ NCs} = \frac{N_A \times (weight\ of\ bisFPA/M_w(bisFPA))}{weight\ of NCs/weight\ of\ a\ single\ NC} = \frac{weight\ of\ bisFPA}{weight\ of\ NCs} \times \frac{N_A \times \rho_{CsPbBr3} \times d^3}{M_w(bisFPA)}$$

, where the mass ratio of bisFPA to NCs is 20 wt.%, $M_w$(bisFPA) is 496 g/mol, $\rho(CsPbBr_3)$ = 4.42 g cm$^{-3}$, $d$ = 7.7 nm. The ratio between the numbers of bisFPA and oleyl molecules or the sum of oleyl molecules and ODE can be calculated accordingly.

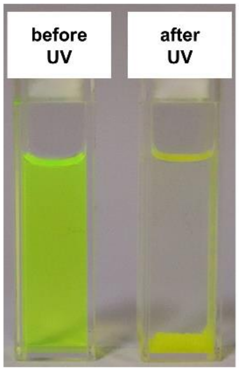


**Fig. S1. Photographs of $CsPbBr_3$ NCs in toluene with bisFPA additives before and after UV exposure.** NCs lose colloidal stability after UV exposure due to the crosslinking of surface ligands.

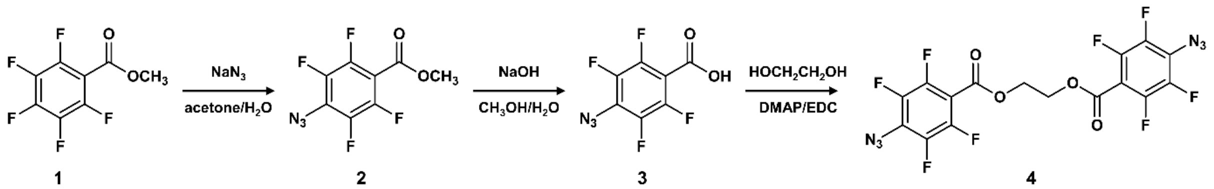


**Fig. S2. Synthesis of the bisFPA photocrosslinker.** Ethylene bis(4-azido-2,3,5,6-tetrafluorobenzoate).

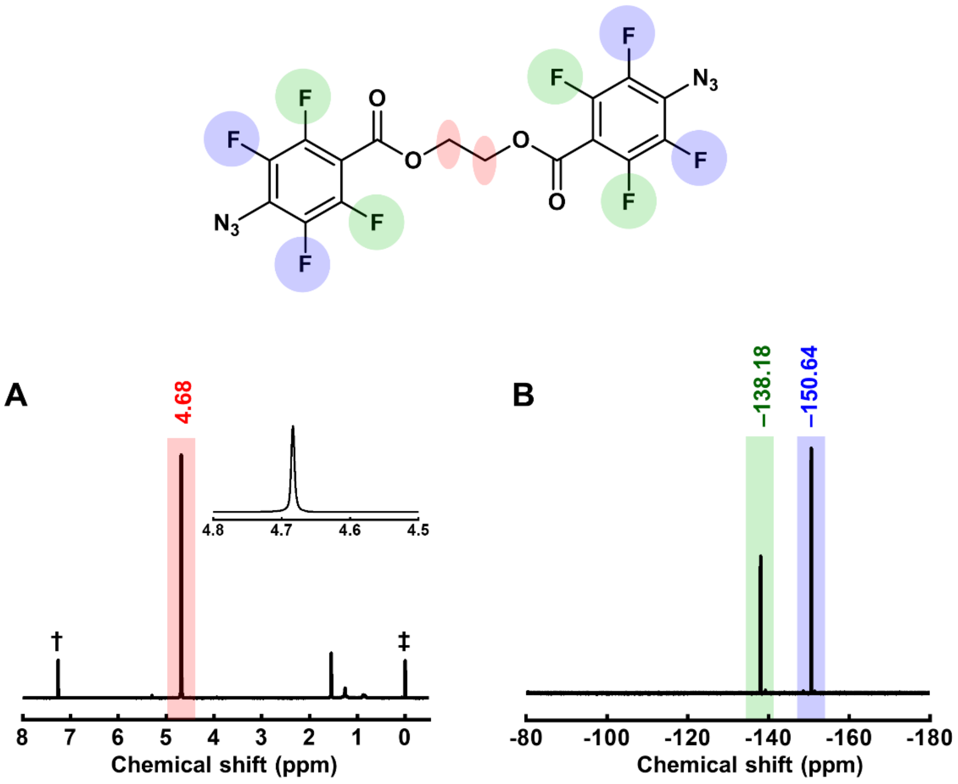


**Fig. S3. NMR spectra of bisFPA in $CDCl_3$. (A)** $^1H$ and **(B)** $^{19}F$-NMR spectra. The color shades indicate $^1H$ and $^{19}F$ resonances corresponding to those marked in the chemical structure of bisFPA. Labeled are the resonance of (†) solvent and (‡) TMS.

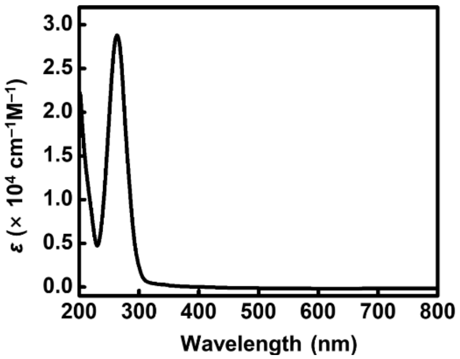


**Fig. S4. Molar extinction coefficient spectrum of bisFPA in acetonitrile.**

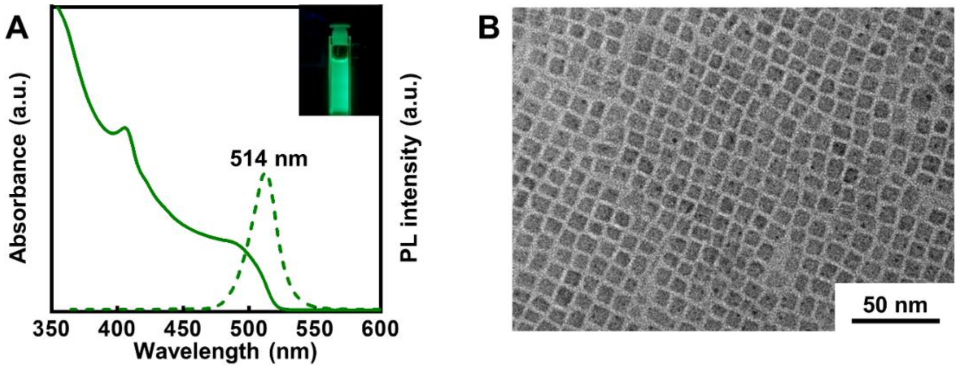


**Fig. S5. The absorbance and PL spectra and TEM image of $CsPbBr_3$ NCs. (A)** The absorbance (solid) and PL (dashed) spectra and **(B)** TEM image of $CsPbBr_3$ NCs.

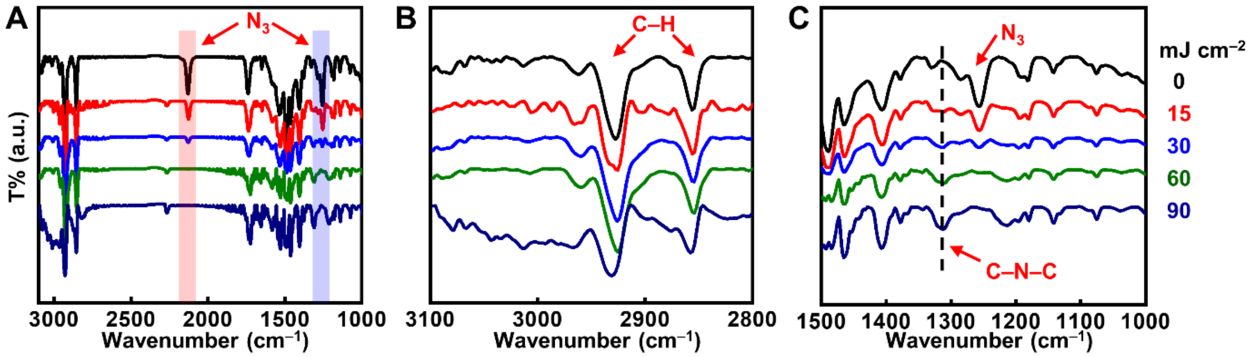


**Fig. S6. FTIR spectra of thin films composed of $CsPbBr_3$ NCs and bisFPA with different UV doses. (A)** The FTIR spectra of thin films with different UV doses (from top to bottom, 0, 15, 30, 60 and 90 mJ cm$^{-2}$ at 254 nm), highlighting changes in the intensity of peaks corresponding to –$N_3$ moiety. Asymmetric (2130 cm$^{-1}$) and symmetric (1250 cm$^{-1}$) vibrational modes are highlighted by red and blue shades. The absorption peaks at ~2300 cm$^{-1}$ in some of the curves are probably from residual $CO_2$. **(B, C)** FTIR spectra in the range of 3100–2800 and 1500–1000 cm$^{-1}$, respectively, highlighting the vibrational modes of C–H, –$N_3$, and C–N–C bonds.

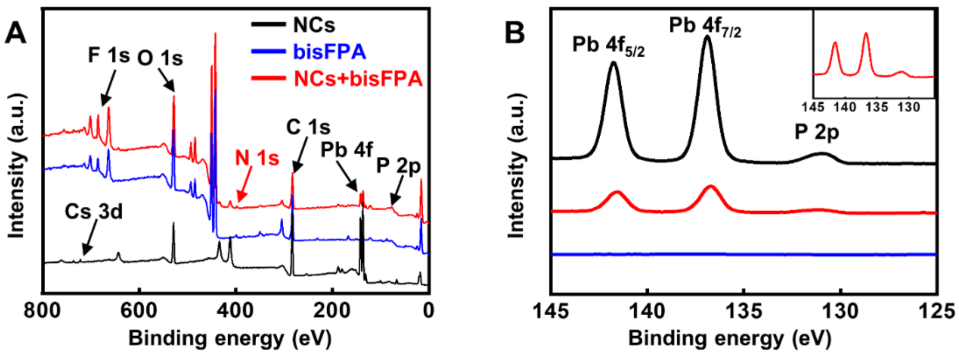


**Fig. S7. XPS spectra of thin films of OPA-capped $CsPbBr_3$ NCs, bisFPA, and their mixture after UV exposure and developing. (A)** The XPS survey spectra. **(B)** XPS spectra highlighting Pb 4f and P 2p regions. Inset in (B) is the maginified view of spectrum for thin films of the mixture of NCs and bisFPA after exposure and developing.

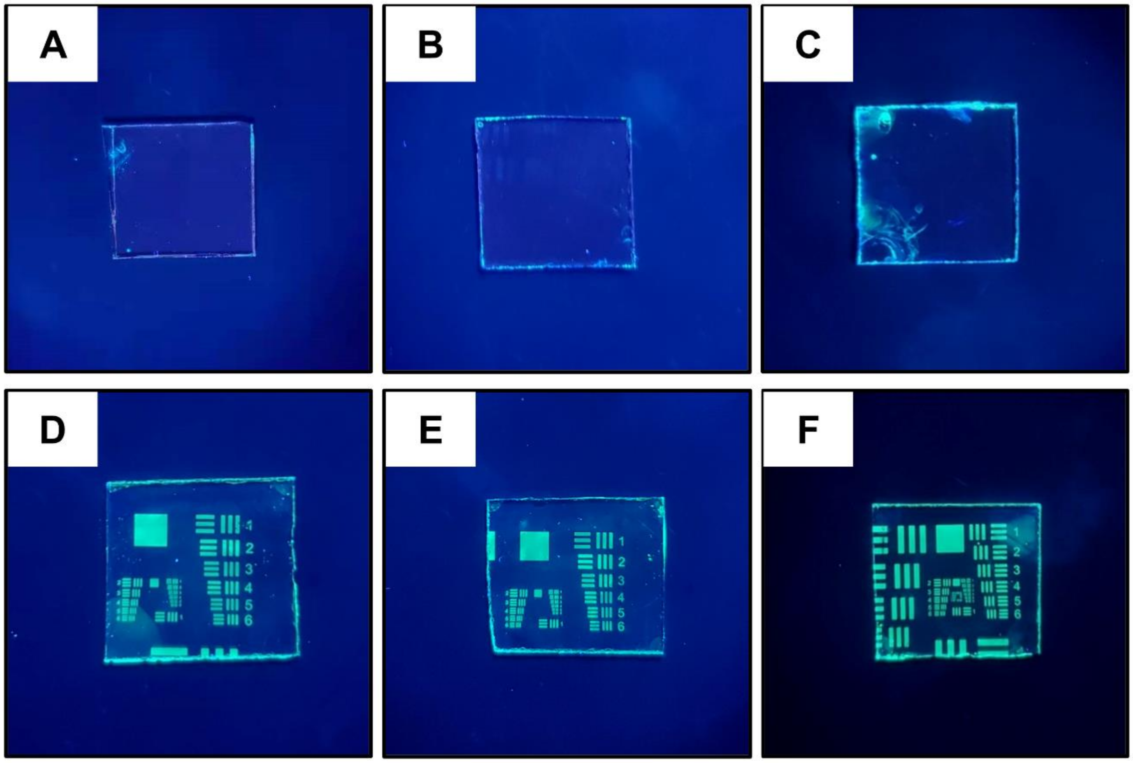


**Fig. S8. Photographs of patterns obtained from $CsPbBr_3$ NCs with different purification procedures. (A–C)** Purified once with MeOAc:NC solution (vol:vol) = 1:1, 2:1, and 3:1, respectively. **(D–F)** (A–C) with an additional cycle of purification with MeOAc (MeOAc:NC solution (vol:vol) = 1:1). In all cases, the NC inks contain 20 wt.% bisFPA and the UV dose is 60 mJ $cm^{-2}$. The photomask used here has the form of 1951 US Air Force Target, a standard for evaluating patterning quality.

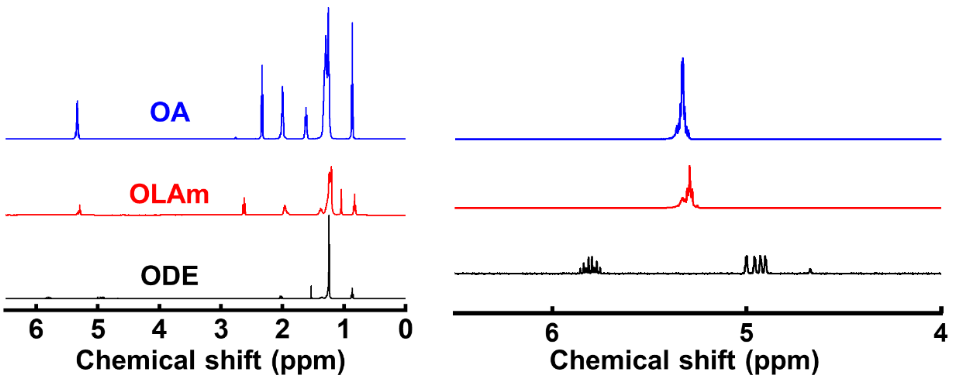


**Fig. S9. $^{1}$H-NMR of ODE, OLAm and OA.** The right panel highlights resonance corresponding to the alkene protons in these molecules.

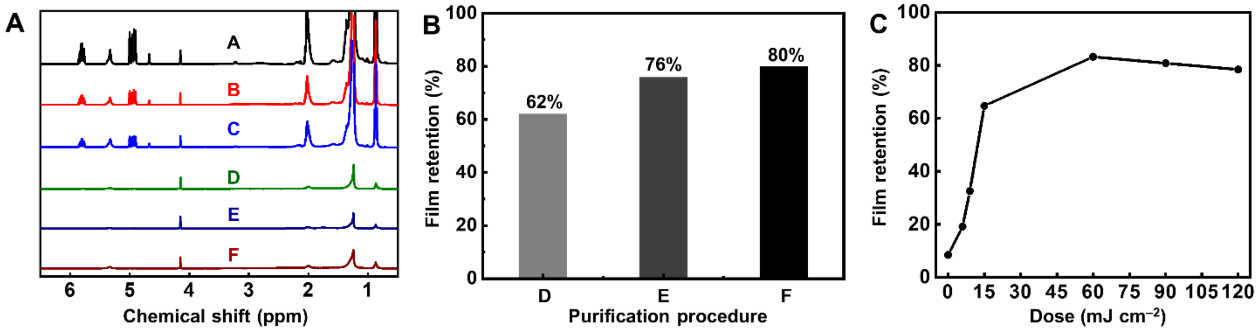


**Fig. S10. Evaluation of the effect of purification procedures and UV doses on the patterning quality. (A)** $^1$H-NMR of $CsPbBr_3$ NCs purified with different procedures. Procedures A to F are the same with those defined in Fig. S8. The resonance at 4.15 ppm is from ferrocene and used as an internal standard. **(B)** Film retention of photocrosslinked thin films starting from $CsPbBr_3$ NCs purified with procedures D, E, and F. In all cases, the NC inks contain 20 wt.% bisFPA and the UV dose is 60 mJ cm$^{-2}$. The exposed thin films were developed with toluene. **(C)** Film retention of photocrosslinked NC thin films versus UV doses.

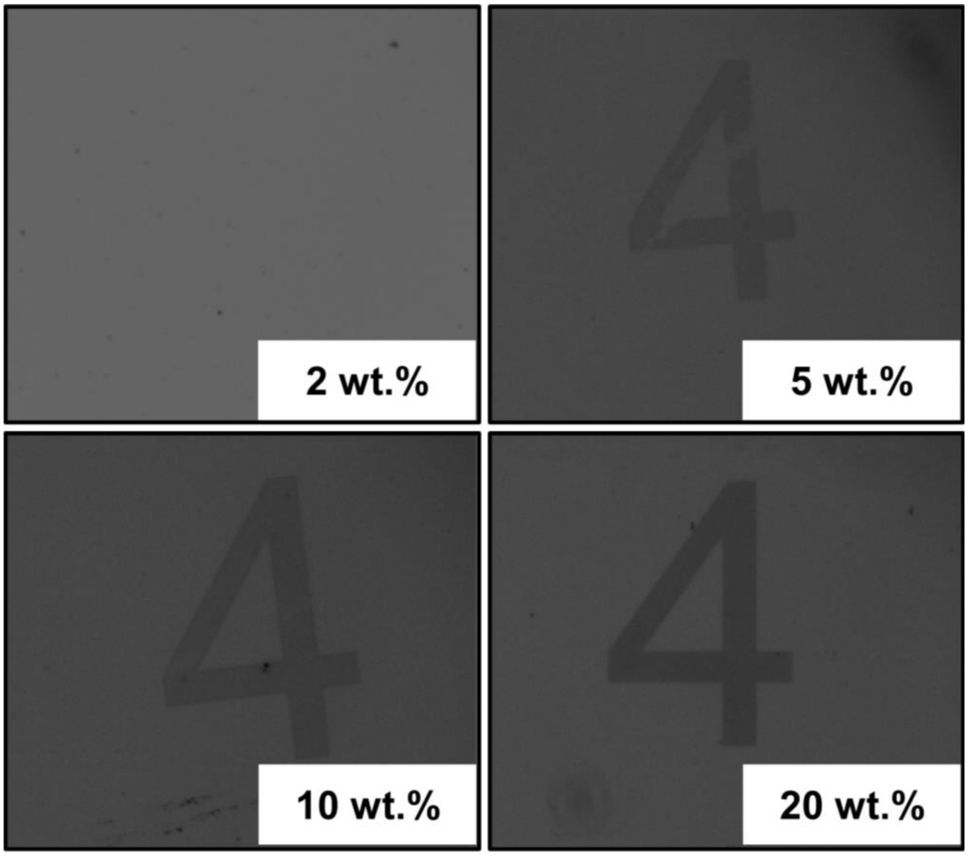


**Fig. S11. Photographs of patterns obtained from $CsPbBr_3$ NCs with different mass ratios of bisFPA.**

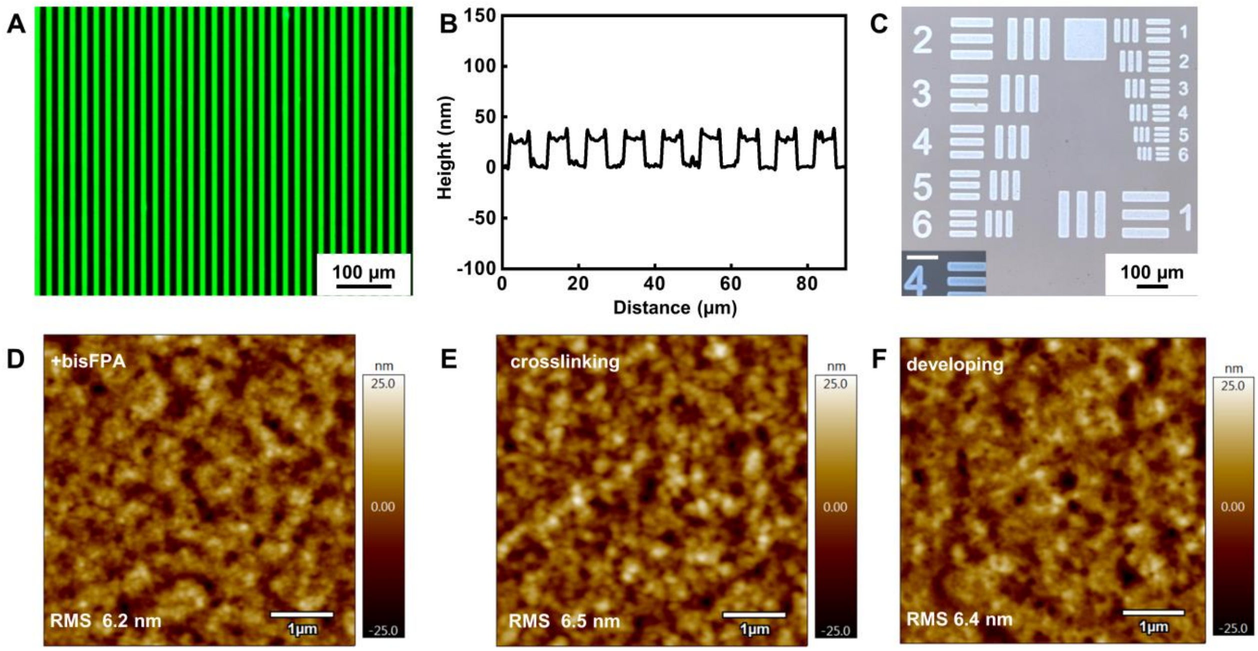


**Fig. S12. Additional microscopic images of $CsPbBr_3$ NC thin film patterns. (A)** Fluorescence optical image of line patterns of $CsPbBr_3$ NC thin films and **(B) t**he corresponding height profile. **(C)** Optical microscopic image of NC thin film patterned in the format of 1951 US Air Force target. Scale bar in inset of (C) is 100 µm. **(D–F)** AFM images of NC thin films (D) before crosslinking, (E) after crosslinking and (F) after solvent developing.

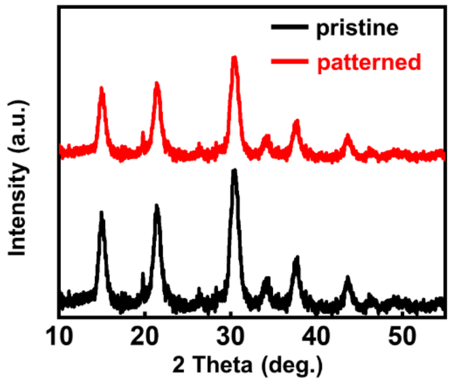


**Fig. S13. XRD patterns of pristine $CsPbBr_3$ NC thin films and after patterning via DOPPLCER.**

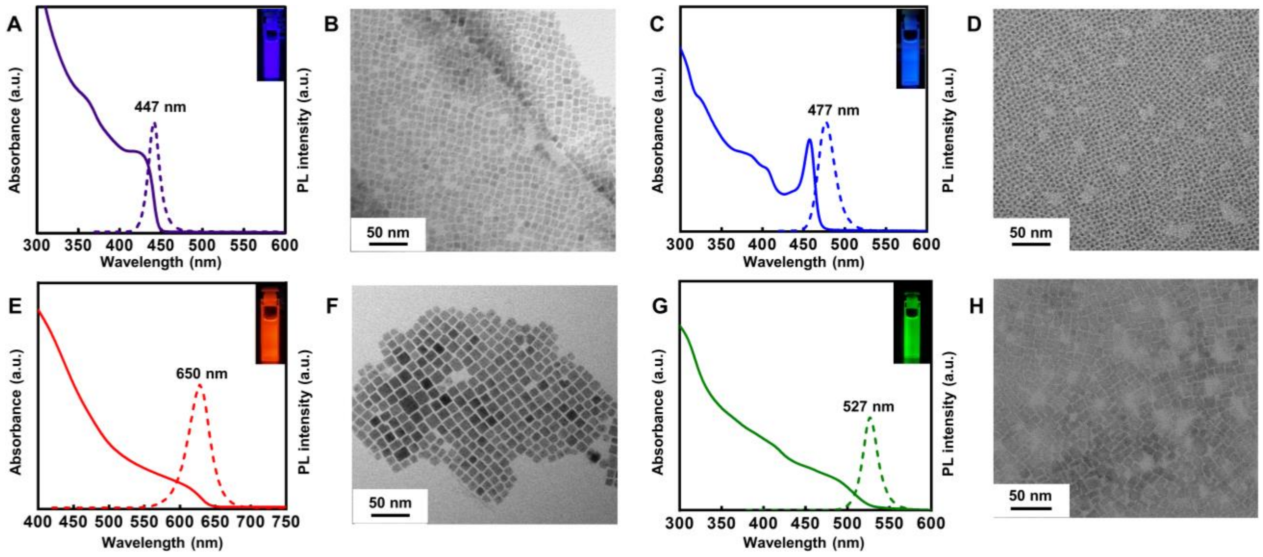


**Fig. S14. UV-vis and PL spectra and TEM images of various perovskite NCs. (A,B)** $CsPbCl_{3-x}Br_x$ NCs; **(C,D)** blue-emitting $CsPbBr_3$ QDs; **(E,F)** $CsPbI_{3-x}Br_x$ NCs; **(G,H)** $FAPbBr_3$ NCs synthesized via LARP method.

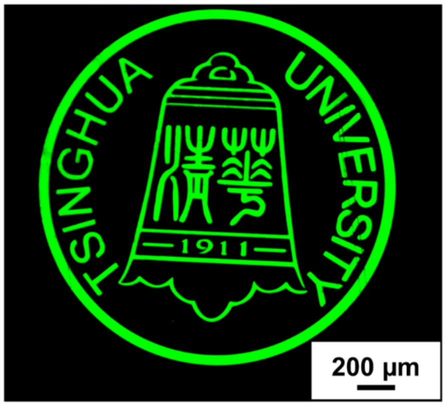


**Fig. S15. Fluorescence optical microscopic image of patterned $CsPbBr_3$ NCs with zwitterionic ligands.**

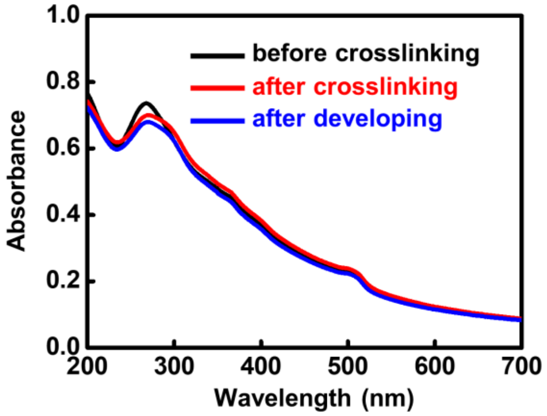


**Fig. S16. The UV-vis absorption spectra of spin-coated thin films containing $CsPbBr_3$ NCs and bisFPA at different stages of DOPPLCER.** The mass ratio of bisFPA to NCs is 20 wt.%. The slight changes in the absorption around 260 nm may be related to the photolysis of bisFPA during crosslinking and the extraction of unreacted bisFPA by developer solvent (*33*).

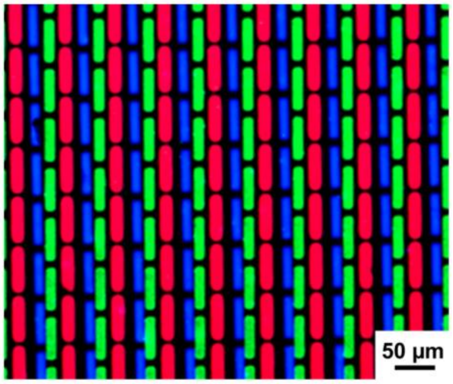


**Fig. S17. Fluorescence optical microscopic image of full-colored pixelated patterns of RGB NCs.** R: II-VI QDs, G: $CsPbBr_3$ NCs, B: $CsPbBr_3$ QDs.

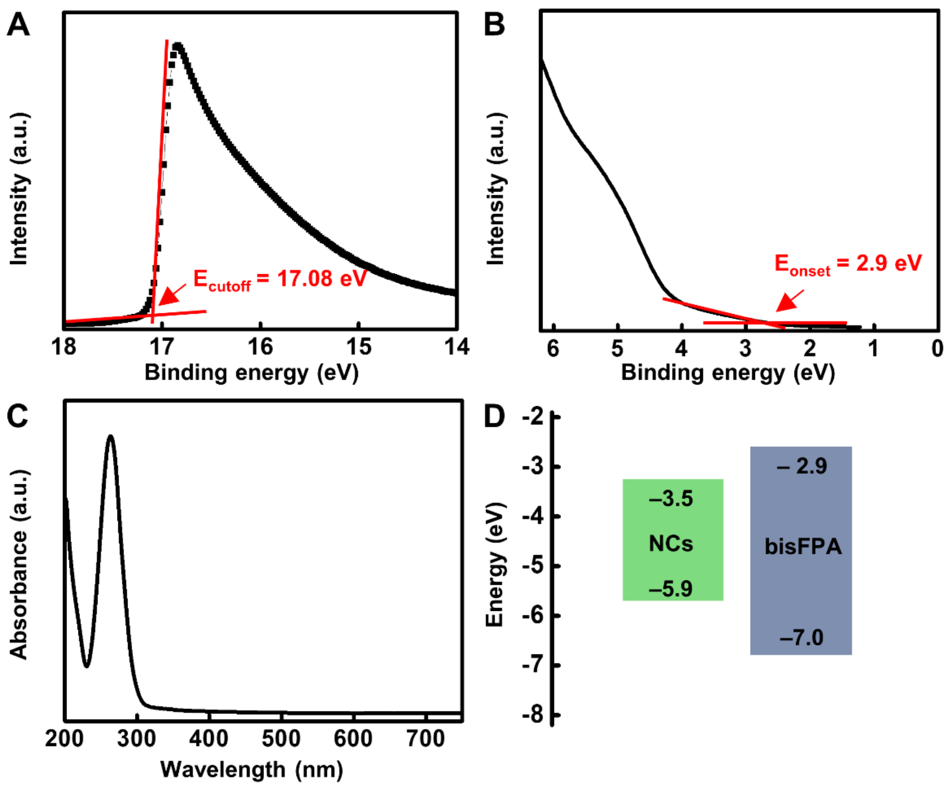


**Fig. S18. UPS spectra and energy band levels of bisFPA. (A,B)** UPS spectra of bisFPA in (A) the secondary edge region and (B) the HOMO region. **(C)** UV-vis absorption spectrum of bisFPA. **(D)** Calculated energy band levels of bisFPA and $CsPbBr_3$ NCs. The energy levels (versus vacuum) for $CsPbBr_3$ NCs are from ref. (*56*).

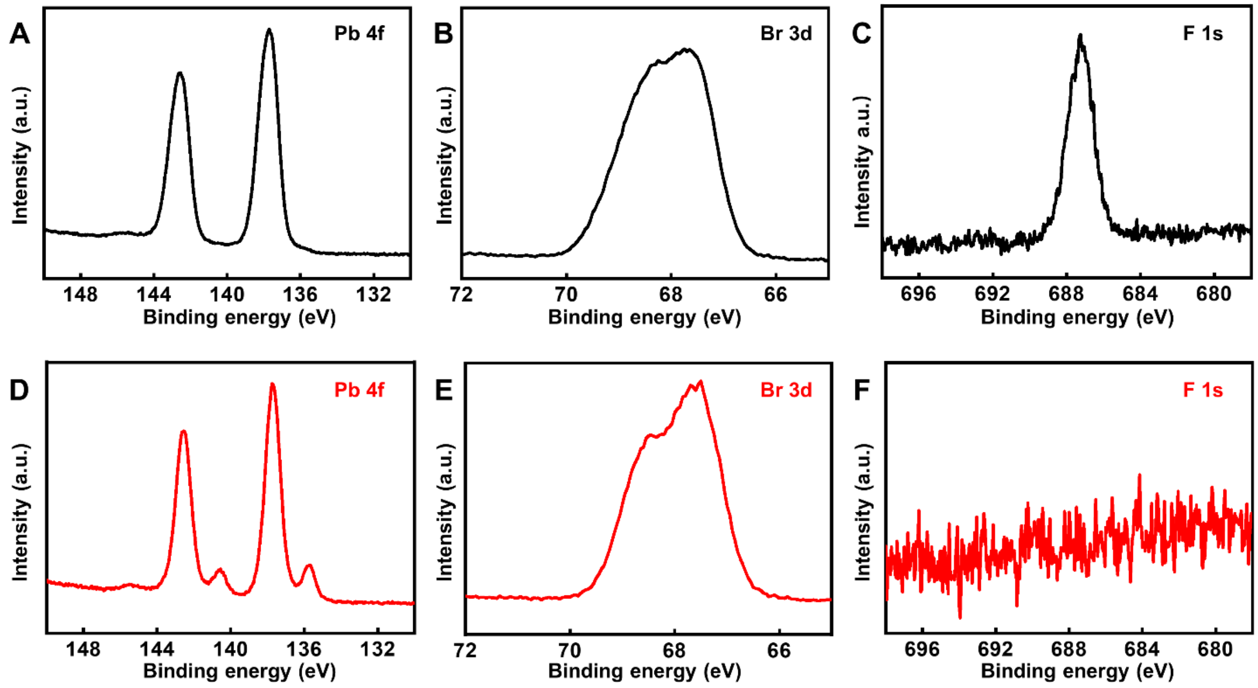


**Fig. S19. XPS spectra of DOPPLCER-treated $CsPbBr_3$ NC thin films before and after post-patterning treatment with $PbBr_2$/OLAm/OA**. Data in the regions of Pb 4f, Br 3d and F 1s for samples **(A–C)** before and **(D–F)** after post-patterning treatment. The mass ratio of bisFPA to NCs is 20 wt.% and the UV dose is 60 mJ $cm^{-2}$.

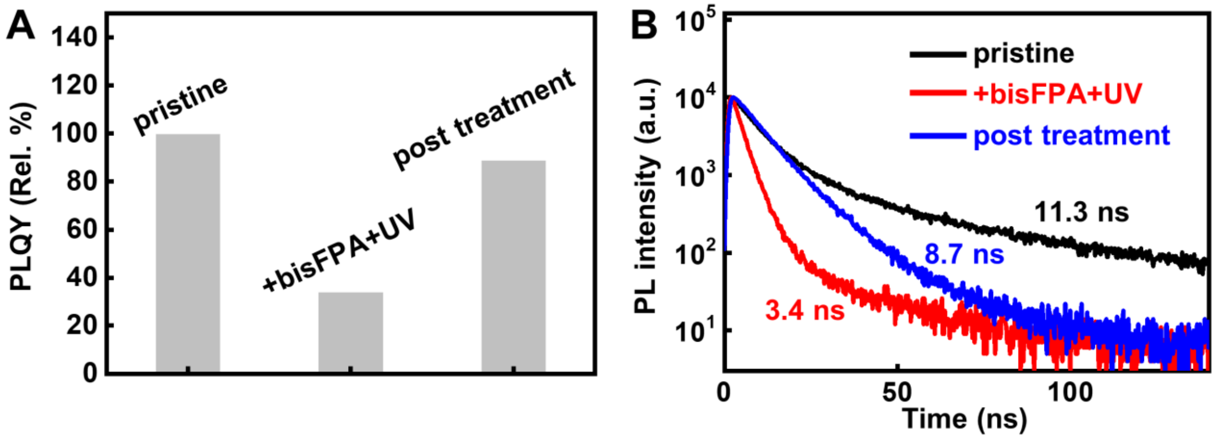


**Fig. S20. PL characteristics of thin films of zwitterionic ligand capped $CsPbBr_3$ NCs. (A)** Relative PLQY before (pristine) and after (+bisFPA+UV) patterning and after post-treatment (brief soaking in a mixture of $PbBr_2$/OLAm/OA). **(B)** Corresponding PL decay curves and calculated averaged lifetime of film samples.

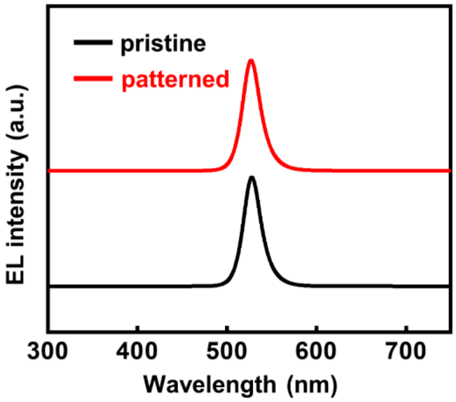


**Fig. S21. EL emission spectra of pristine and patterned $FAPbBr_3$ NC LEDs.**

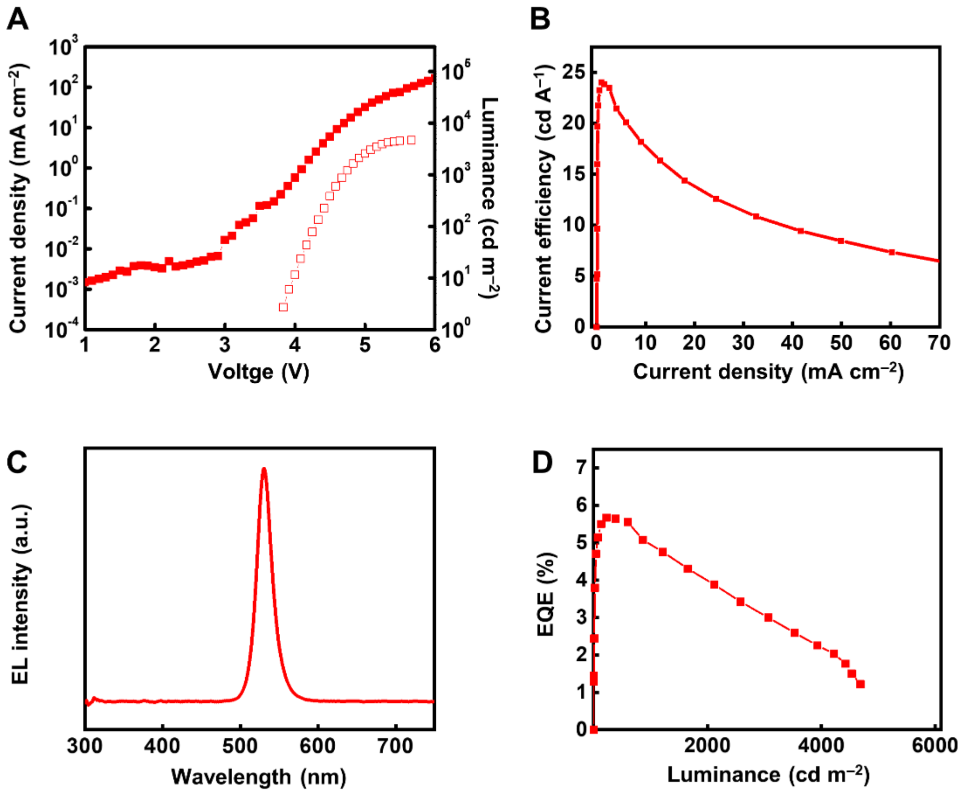


**Fig. S22. EL characteristics of LEDs processed by DOPPLCER with one-layer patterned $FAPbBr_3$ NCs. (A)** Current density–voltage–luminance (*J*–*V*–*L*), **(B)** Current efficiency–current density (*CE*–*J*), **(C)** EL emission spectrum and **(D)** EQE of devices.

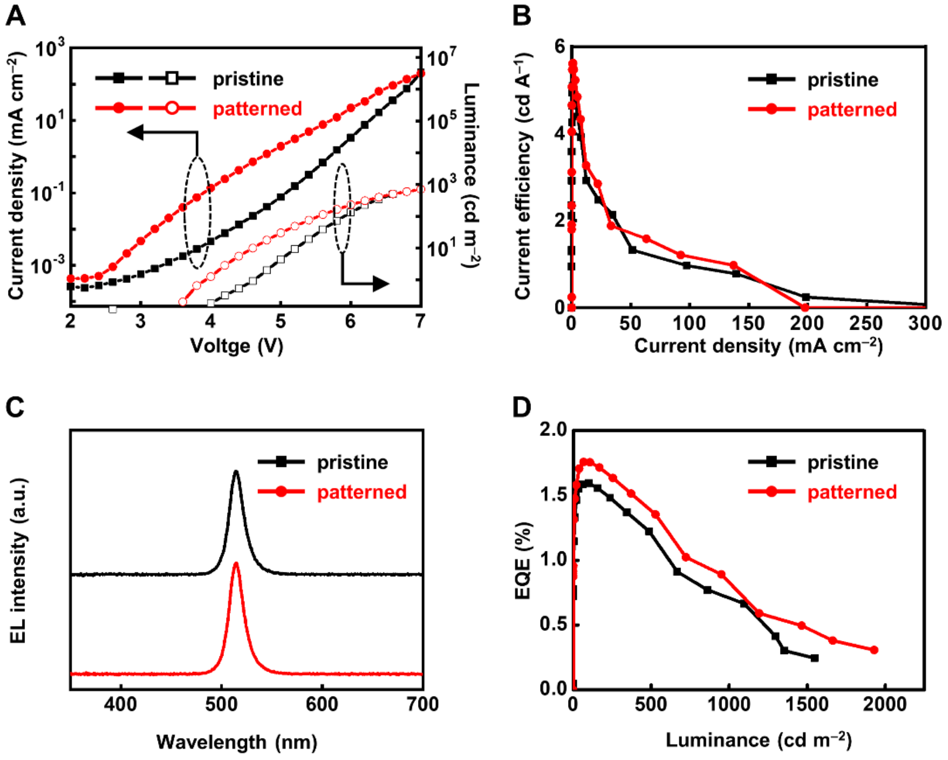


**Fig. S23. EL characteristics of $CsPbBr_3$ NC LEDs processed by DOPPLCER. (A)** Current density–voltage–luminance (*J*–*V*–*L*), **(B)** Current efficiency–current density (*CE*–*J*), **(C)** EL spectrum and **(D)** EQE characteristics of pristine and patterned devices. The mass ratio of bisFPA is 20 wt.% and the UV dose is 60 mJ $cm^{-2}$.

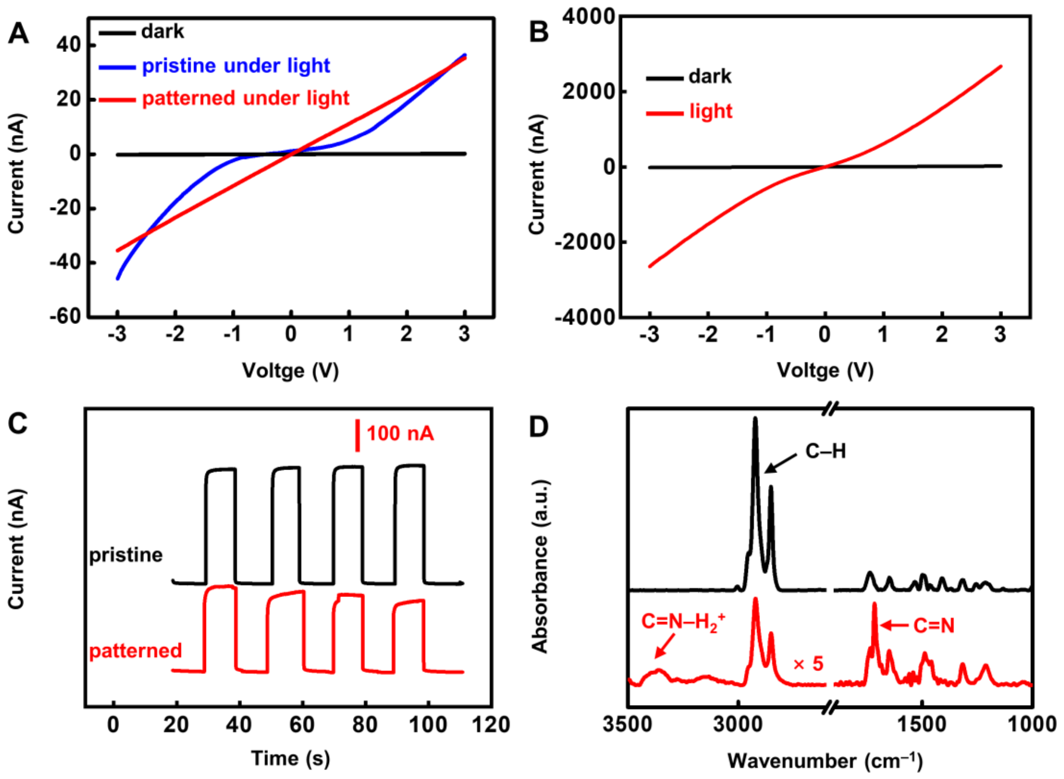


**Fig. S24. Photocurrent–voltage curves and photocurrent–time response of prototype $CsPbBr_3$ photoconductors processed by DOPPLCER. (A)** photo- and dark current of pristine (blue line) and patterned (red line) $CsPbBr_3$ NCs without ligand exchange. **(B)** photo- and dark current of patterned $CsPbBr_3$ NCs after ligand exchange. The devices were illuminated by 450 nm light with intensity of 13.0 mW cm$^{-2}$. **(C)** Photocurrent–time response of pristine and patterned $CsPbBr_3$ NC thin films under dark and illumination (450 nm, 0.84 mW cm$^{-2}$) conditions with an applied voltage of 3 V. Both pristine and patterned films were ligand-exchanged following reported procedures. **(D)** FTIR spectra of patterned $CsPbBr_3$ films before (black line) and after (red line) ligand exchange.

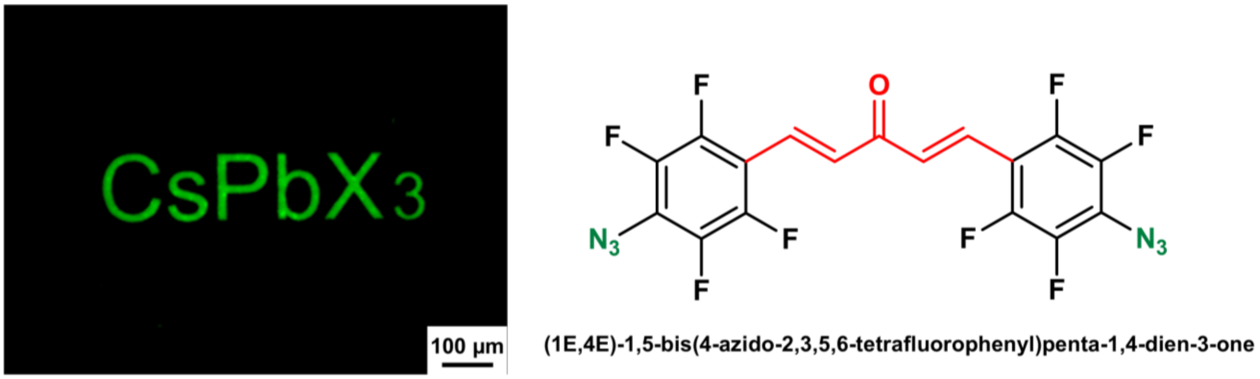


**Fig. S25. Fluorescence optical microscopic image of $CsPbBr_3$ NCs with 365-nm sensitive bisFPA.** Chemical structure shown on the right panel.

**Table S1. Summary of ligand density and ligand/photocrosslinker ratios for $CsPbBr_3$ NCs with different purification procedures.** PP is the abbreviation of purification procedures that were defined in the Materials and Methods. Details in calculation appears in Supplementary Texts.

| PP | Oleyl (mM) | ODE/ Oleyl | $A_{335}$ | NCs (µM) | Ligand density ($nm^{-2}$) | BisFPA/ oleyl | BisFPA/ (oleyl+ODE) |
|---|---|---|---|---|---|---|---|
| A | 3.93 | 1.4 | 0.95 | 3.01 | 3.7 | 0.38 | 0.16 |
| B | 3.91 | 1.1 | 1.06 | 3.36 | 3.3 | 0.42 | 0.20 |
| C | 2.90 | 0.6 | 1.69 | 5.36 | 1.5 | 0.90 | 0.56 |
| D | 0.31 | 0.2 | 0.56 | 1.77 | 0.5 | 2.80 | 2.37 |
| E | 0.22 | 0.2 | 0.47 | 1.48 | 0.4 | 3.34 | 2.74 |
| F | 0.70 | 0.07 | 0.98 | 3.10 | 0.6 | 2.17 | 2.03 |

**Table S2. Comparison of the absolute PLQYs of $CsPbBr_3$ and $FAPbBr_3$ NC thin films with different treatments involved in DOPPLCER and after post-patterning treatment.**

| Samples | Pristine | +UV | +bisFPA | +UV+bisFPA | Post-treated |
|---|---|---|---|---|---|
| $CsPbBr_3$ | 61% | 52% | 58% | 39% | 76% |
| $FAPbBr_3$ | 86% | 67% | 82% | 48% | 75% |

**Table S3. Fitting parameters for time-resolved PL of $CsPbBr_3$ NC thin films with different treatments involved in DOPPLCER and after post-patterning treatment.**

| Samples | A | $B_1$ (%) | $\tau_1$ (ns) | $B_2$ (%) | $\tau_2$ (ns) | $\tau_{avg}$ (ns) |
|---|---|---|---|---|---|---|
| pristine | 11.3 | 71.0 | 5.5 | 28.0 | 50.0 | 7.5 |
| +UV | 10.1 | 69.6 | 5.2 | 30.4 | 40.9 | 7.1 |
| +bisFPA | 7.7 | 74.3 | 5.2 | 25.7 | 33.6 | 6.6 |
| +UV+bisFPA | 8.4 | 72.3 | 3.4 | 27.7 | 32.4 | 4.6 |
| post-treated | 14.0 | 80.1 | 8.6 | 19.9 | 46.3 | 10.2 |

$B_1$ and $B_2$ are parameters in the bi-exponential fitting of the time-resolved PL and presented in the form of weighted percentage of the two decays. The fitting follows $y = A + B_1 e^{\left(-\frac{x}{\tau_1}\right)} + B_2 e^{\left(-\frac{x}{\tau_2}\right)}$. The average PL lifetime ($\tau_{avg}$) was provided by the commercial software for the TCSPC toolkit on Horiba FluoroMax Plus spectrometer.

**Table S4. Fitting parameters for time-resolved PL of $FAPbBr_3$ NC thin films with different treatments involved in DOPPLCER.**

| Samples | A | $B_1$ (%) | $\tau_1$ (ns) | $B_2$ (%) | $\tau_2$ (ns) | $\tau_{avg}$ (ns) |
|---|---|---|---|---|---|---|
| pristine | 12.0 | 78.1 | 15.2 | 21.9 | 44.1 | 17.7 |
| +UV | 11.0 | 79.7 | 14.1 | 20.3 | 39.9 | 16.2 |
| +bisFPA | 14.5 | 74.9 | 15.1 | 25.1 | 43.7 | 18.1 |
| +UV+bisFPA | 11.3 | 74.2 | 11.1 | 25.8 | 30.2 | 13.3 |

$B_1$ and $B_2$ are parameters in the bi-exponential fitting of the time-resolved PL and presented in the form of weighted percentage of the two decays. The fitting follows $y = A + B_1 e^{\left(-\frac{x}{\tau_1}\right)} + B_2 e^{\left(-\frac{x}{\tau_2}\right)}$. The average PL lifetime ($\tau_{avg}$) was provided by the commercial software for the TCSPC toolkit on Horiba FluoroMax Plus spectrometer.

**Table S5. Summary of device characteristics of pristine and patterned LEDs with $CsPbBr_3$ and $FAPbBr_3$ NCs.**

| Devices | $CE_{max}$ (cd $A^{-2}$) | $EQE_{max}$ (%) | $L_{max}$ (cd $m^{-2}$) | $V_{turn-on}$ (V) |
|---|---|---|---|---|
| $FAPbBr_3$ pristine | 25.9 | 6.1 | 9582 | 3.2 |
| 1-layer patterned | 24.0 | 5.7 | 4684 | 3.2 |
| 2-layer patterned | 28.5 | 6.8 | 20900 | 3.3 |
| $CsPbBr_3$ pristine | 5.1 | 1.6 | 1547 | 3.6 |
| $CsPbBr_3$ patterned | 5.6 | 1.8 | 1929 | 3.6 |

**Table S6. Summary of device characteristics of perovskite LEDs with different patterning methods.**

| Active layer | Patterning methods | $CE_{max}$ (cd $A^{-2}$) | $EQE_{max}$ (%) | $L_{max}$ (cd $m^{-2}$) | $V_{turn-on}$ (V) |
|---|---|---|---|---|---|
| $FAPbBr_3$ NCs | DOPPLCER (this work) | 28.5 | 6.8 | 20900 | 3.3 |
| $MAPbBr_3$ NCs | crosslinkable ligands (*30*) | 6.7 | N/A | 9700 | 4.5 |
| $CsPbBr_3$ NCs | ink-jet printing (*21*) | 10.3 | 3.4 | 956 | 3.2 |
| $FA_{0.3}Cs_{0.7}PbBr_3$ NCs | ink-jet printing (*20*) | 10.3 | 2.8 | 1233 | 3.0 |
| $(PEABr)_{0.4}CsPbBr_3$ quasi-2D films | Photo-lithography (*57*) | 3.85 | 1.24 | 13043 | 3.4 |
| $MAPbI_3$ films | PDMS molding (*58*) | N/A | 0.012 | 0.53* | N/A |
| $CsPbI_3$ crystals | confined growth (*59*) | N/A | N/A | 0.012 | N/A |
| $MAPbBr_3$ single crystals | homoepitaxial growth (*60*) | N/A | 6.1 | N/A | <3.0 |

* The unit is W $sr^{-1}$ $m^{-2}$

**Table S7. Comparison of different patterning methods for perovskite NCs.**

| Methods | Resolution | Versatility | Compatibility with Devices | Comments |
| --- | --- | --- | --- | --- |
| DOPPLCER (this work) | ~5 μm* | various NCs and ligands | High | NCs with native ligands; multicolored patterning via anion exchange or with proper choices of NCs |
| DOLFIN (*28*) | ~1 μm | $CsPbX_3$ NCs after ligand exchange | High | photocleavable ligands; multicolored patterning via anion exchange |
| photo-crosslinkable ligands (*29-31*) | ~5 μm | $CsPbX_3$ or $MAPbX_3$ NCs after ligand exchange | High | photocrosslinkable ligands; high contents of insulating polymeric ligands or matrices; multicolored patterning |
| photolitho-graphy (*24*) | ~1 μm | $CsPbBr_3$ NCs | Moderate | complicated fabrication steps; inconvenient for LEDs |
| ink-jet printing (*18, 20, 61*) | >50 μm | pre-synthesized or in-situ NC formation | High | limited resolution and issues in film quality; usually involve insulating polymers; easy to achieve multicolored patterning |
| electro-hydrodynamic jet printing (*62*) | ~5 μm | in-situ NC formation | Moderate | complexities in ink compositions and printing parameters |
| other printing methods (*22, 23*) | tens of nm | $CsPbX_3$ NCs or in-situ formation of single crystals | Moderate | use of unconventional apparatus and chemically prepatterned substrates by high-voltage writing |
| X-ray lithography (*15, 16*) | ~100 nm | $CsPbX_3$ NCs | Low | constraints in apparatus and conditions; destructive to NCs with severe PL degradation |
| direct laser writing (*17*) | ~10 μm | $CsPbBr_3$ NCs | Low | low resolution; destructive to NCs such as sintering and degradation |

* The resolution was limited by those of photo masks and mask aligners.

## REFERENCES AND NOTES

1. M. V. Kovalenko, L. Protesescu, M. I. Bodnarchuk, Properties and potential optoelectronic applications of lead halide perovskite nanocrystals. *Science* **358**, 745–750 (2017).

2. H. Huang, L. Polavarapu, J. A. Sichert, A. S. Susha, A. S. Urban, A. L. Rogach, Colloidal lead halide perovskite nanocrystals: Synthesis, optical properties and applications. *NPG Asia Mater.* **8**, e328 (2016).

3. G. Almeida, I. Infante, L. Manna, Resurfacing halide perovskite nanocrystals. *Science* **364**, 833–834 (2019).

4. J. Shamsi, A. S. Urban, M. Imran, L. De Trizio, L. Manna, Metal halide perovskite nanocrystals: Synthesis, post-synthesis modifications, and their optical properties. *Chem. Rev.* **119**, 3296–3348 (2019).

5. A. Swarnkar, V. K. Ravi, A. Nag, Beyond colloidal cesium lead halide perovskite nanocrystals: Analogous metal halides and doping. *ACS Energy Lett.* **2**, 1089–1098 (2017).

6. S. G. Motti, F. Krieg, A. J. Ramadan, J. B. Patel, H. J. Snaith, M. V. Kovalenko, M. B. Johnston, L. M. Herz, $CsPbBr_3$ nanocrystal films: Deviations from bulk vibrational and optoelectronic properties. *Adv. Funct. Mater.* **30**, 1909904 (2020).

7. Y. Dong, Y.-K. Wang, F. Yuan, A. Johnston, Y. Liu, D. Ma, M.-J. Choi, B. Chen, M. Chekini, S.-W. Baek, L. K. Sagar, J. Fan, Y. Hou, M. Wu, S. Lee, B. Sun, S. Hoogland, R. Quintero-Bermudez, H. Ebe, P. Todorovic, F. Dinic, P. Li, H. Kung, M. I. Saidaminov, E. Kumacheva, E. Spiecker, L.-S. Liao, O. Voznyy, Z.-H. Lu, E. H. Sargent, Bipolar-shell resurfacing for blue LEDs based on strongly confined perovskite quantum dots. *Nat. Nanotechnol.* **15**, 668–674 (2020).

8. Y.-H. Kim, S. Kim, A. Kakekhani, J. Park, J. Park, Y.-H. Lee, H. Xu, S. Nagane, R. B. Wexler, D.-H. Kim, S. H. Jo, L. Martinez-Sarti, P. Tan, A. Sadhanala, G.-S. Park, Y.-W. Kim, B. Hu, H. J. Bolink, S. Yoo, R. H. Friend, A. M. Rappe, T.-W. Lee, Comprehensive defect suppression in perovskite nanocrystals for high-efficiency light-emitting diodes. *Nat. Photonics* **15**, 148–155 (2021).

9. A. Swarnkar, A. R. Marshall, E. M. Sanehira, B. D. Chernomoridik, D. T. Moore, J. A. Christians, T. Chakrabarti, J. M. Luther, Quantum dot-induced phase stabilization of α-$CsPbI_3$ perovskite for high-efficiency photovoltaics. *Science* **354**, 92–95 (2016).

10. E. M. Sanehira, A. R. Marshall, J. A. Christians, S. P. Harvey, P. N. Ciesielski, L. M. Wheeler, P. Schulz, L. Y. Lin, M. C. Beard, J. M. Luther, Enhanced mobility $CsPbI_3$ quantum dot arrays for record-efficiency, high-voltage photovoltaic cells. *Sci. Adv.* **3**, eaao4204 (2017).

11. M. Gandini, I. Villa, M. Beretta, C. Gotti, M. Imran, F. Carulli, E. Fantuzzi, M. Sassi, M. Zaffalon, C. Brofferio, L. Manna, L. Beverina, A. Vedda, M. Fasoli, L. Gironi, S. Brovelli, Efficient, fast and reabsorption-free perovskite nanocrystal-based sensitized plastic scintillators. *Nat. Nanotechnol.* **15**, 462–468 (2020).

12. S. Yakunin, L. Protesescu, F. Krieg, M. I. Bodnarchuk, G. Nedelcu, M. Humer, G. De Luca, M. Fiebig, W. Heiss, M. V. Kovalenko, Low-threshold amplified spontaneous emission and lasing from colloidal nanocrystals of caesium lead halide perovskites. *Nat. Commun.* **6**, 8056–8064 (2015).

13. H. Utzat, W. Sun, A. E. K. Kaplan, F. Krieg, M. Ginterseder, B. Spokoyny, N. D. Klein, K. E. Shulenberger, C. F. Perkinson, M. V. Kovalenko, M. G. Bawendi, Coherent single-photon emission from colloidal lead halide perovskite quantum dots. *Science* **363**, 1068–1072 (2019).

14. B. Jeong, H. Han, C. Park, Micro-and nanopatterning of halide perovskites where crystal engineering for emerging photoelectronics meets integrated device array technology. *Adv. Mater.* **32**, 2000597 (2020).

15. F. Palazon, M. Prato, L. Manna, Writing on nanocrystals: Patterning colloidal inorganic nanocrystal films through irradiation-induced chemical transformations of surface ligands. *J. Am. Chem. Soc.* **139**, 13250–13259 (2017).

16. F. Palazon, Q. A. Akkerman, M. Prato, L. Manna, X-ray lithography on perovskite nanocrystals films: From patterning with anion-exchange reactions to enhanced stability in air and water. *ACS Nano* **10**, 1224–1230 (2016).

17. J. Chen, Y. Wu, X. Li, F. Cao, Y. Gu, K. Liu, X. Liu, Y. Dong, J. Ji, H. Zeng, Simple and fast patterning process by laser direct writing for perovskite quantum dots. *Adv. Mater. Technol.* **2**, 1700132 (2017).

18. Y. Liu, F. Li, L. Qiu, K. Yang, Q. Li, X. Zheng, H. Hu, T. Guo, C. Wu, T. W. Kim, Fluorescent microarrays of in situ crystallized perovskite nanocomposites fabricated for patterned applications by using inkjet printing. *ACS Nano* **13**, 2042–2049 (2019).

19. L. Shi, L. Meng, F. Jiang, Y. Ge, F. Li, X.-g. Wu, H. Zhong, In situ inkjet printing strategy for fabricating perovskite quantum dot patterns. *Adv. Funct. Mater.* **29**, 1903648 (2019).

20. D. Li, J. Wang, M. Li, G. Xie, B. Guo, L. Mu, H. Li, J. Wang, H. L. Yip, J. Peng, Inkjet printing matrix perovskite quantum dot light-emitting devices. *Adv. Mater. Technol.* **5**, 2000099 (2020).

21. J. Wang, D. Li, L. Mu, M. Li, Y. Luo, B. Zhang, C. Mai, B. Guo, L. Lan, J. Wang, H. L. Yip, J. Peng, Inkjet-printed full-color matrix quasi-two-dimensional perovskite light-emitting diodes. *ACS Appl. Mater. Interfaces* **13**, 41773–41781 (2021).

22. J. S. Du, D. Shin, T. K. Stanev, C. Musumeci, Z. Xie, Z. Huang, M. Lai, L. Sun, W. Zhou, N. P. Stern, V. P. Dravid, C. A. Mirkin, Halide perovskite nanocrystal arrays: Multiplexed synthesis and size-dependent emission. *Sci. Adv.* **6**, eabc4959 (2020).

23. X. Xing, Z. Man, J. Bian, Y. Yin, W. Zhang, Z. Lu, High-resolution combinatorial patterning of functional nanoparticles. *Nat. Commun.* **11**, 6002 (2020).

24. C. H. Lin, Q. Zeng, E. Lafalce, S. Yu, M. J. Smith, Y. J. Yoon, Y. Chang, Y. Jiang, Z. Lin, Z. V. Vardeny, V. V. Tsukruk, Large-area lasing and multicolor perovskite quantum dot patterns. *Adv. Opt. Mater.* **6**, 1800474 (2018).

25. Y. Wang, I. Fedin, H. Zhang, D. V. Talapin, Direct optical lithography of functional inorganic nanomaterials. *Science* **357**, 385–388 (2017).

26. Y. Wang, J.-A. Pan, H. Wu, D. V. Talapin, Direct wavelength-selective optical and electron-beam lithography of functional inorganic nanomaterials. *ACS Nano* **13**, 13917–13931 (2019).

27. H. Cho, J. A. Pan, H. Wu, X. Lan, I. Coropceanu, Y. Wang, W. Cho, E. A. Hill, J. S. Anderson, D. V. Talapin, Direct optical patterning of quantum dot light-emitting diodes via in situ ligand exchange. *Adv. Mater.* **32**, 2003805 (2020).

28. J.-A. Pan, J. C. Ondry, D. V. Talapin, Direct optical lithography of $CsPbX_3$ nanocrystals via photoinduced ligand cleavage with postpatterning chemical modification and electronic coupling. *Nano Lett.* **21**, 7609–7616 (2021).

29. J. Ko, K. Ma, J. F. Joung, S. Park, J. Bang, Ligand-assisted direct photolithography of perovskite nanocrystals encapsulated with multifunctional polymer ligands for stable, full-colored, high-resolution displays. *Nano Lett.* **21**, 2288–2295 (2021).

30. H. Lee, J. W. Jeong, M. G. So, G. Y. Jung, C. L. Lee, Design of chemically stable organic perovskite quantum dots for micropatterned light-emitting diodes through kinetic control of a cross-linkable ligand system. *Adv. Mater.* **33**, 2007855 (2021).

31. H. Kim, N. Hight-Huf, J.-H. Kang, P. Bisnoff, S. Sundararajan, T. Thompson, M. Barnes, R. C. Hayward, T. Emrick, Polymer zwitterions for stabilization of $CsPbBr_3$ perovskite nanoparticles and nanocomposite films. *Angew. Chem. Int. Ed.* **59**, 10802–10806 (2020).

32. S. X. Cai, D. J. Glenn, M. Kanskar, M. Wybourne, J. F. Keana, Development of highly efficient deep-UV and electron beam mediated cross-linkers: Synthesis and photolysis of bis (perfluorophenyl) azides. *Chem. Mater.* **6**, 1822–1829 (1994).

33. S. Shimizu, G. R. Bird, Chemical mechanisms in photoresist systems: I. photochemical cleavage of a bisazide system. *J. Electrochem. Soc.* **124**, 1394 (1977).

34. R.-Q. Png, P.-J. Chia, J.-C. Tang, B. Liu, S. Sivaramakrishnan, M. Zhou, S.-H. Khong, H. S. Chan, J. H. Burroughes, L.-L. Chua, R. H. Friend, P. K. H. Ho, High-performance polymer semiconducting heterostructure devices by nitrene-mediated photocrosslinking of alkyl side chains. *Nat. Mater.* **9**, 152–158 (2010).

35. J. Yang, D. Hahm, K. Kim, S. Rhee, M. Lee, S. Kim, J. H. Chang, H. W. Park, J. Lim, M. Lee, H. Kim, J. Bang, H. Ahn, J. Cho, J. Kwak, B. Kim, C. Lee, W. Bae, M. Kang, High-resolution

patterning of colloidal quantum dots via non-destructive, light-driven ligand crosslinking. *Nat. Commun.* **11**, 2874 (2020).

36. L. Protesescu, S. Yakunin, M. I. Bodnarchuk, F. Krieg, R. Caputo, C. H. Hendon, R. X. Yang, A. Walsh, M. V. Kovalenko, Nanocrystals of cesium lead halide perovskites ($CsPbX_3$, X = Cl, Br, and I): Novel optoelectronic materials showing bright emission with wide color gamut. *Nano Lett.* **15**, 3692–3696 (2015).

37. J. De Roo, M. Ibanez, P. Geiregat, G. Nedelcu, W. Walravens, J. Maes, J. C. Martins, I. V. Driessche, M. V. Kovalenko, Z. Hens, Highly dynamic ligand binding and light absorption coefficient of cesium lead bromide perovskite nanocrystals. *ACS Nano* **10**, 2071–2081 (2016).

38. Y. Tan, Y. Zou, L. Wu, Q. Huang, D. Yang, M. Chen, M. Ban, C. Wu, T. Wu, S. Bai, T. Song, Q. Zhang, B. Sun, Highly luminescent and stable perovskite nanocrystals with octylphosphonic acid as a ligand for efficient light-emitting diodes. *ACS Appl. Mater. Interfaces* **10**, 3784–3792 (2018).

39. H. Zhang, K. Dasbiswas, N. B. Ludwig, G. Han, B. Lee, S. Vaikuntanathan, D. V. Talapin, Stable colloids in molten inorganic salts. *Nature* **542**, 328–331 (2017).

40. Y. Yang, H. Qin, X. Peng, Intramolecular entropy and size-dependent solution properties of nanocrystal–ligands complexes. *Nano Lett.* **16**, 2127–2132 (2016).

41. J. Maes, L. Balcaen, E. Drijvers, Q. Zhao, J. De Roo, A. Vantomme, F. Vanhaecke, P. Geiregat, Z. Hens, Light absorption coefficient of $CsPbBr_3$ perovskite nanocrystals. *J. Phys. Chem. Lett.* **9**, 3093–3097 (2018).

42. Y. Yuan, H. Zhu, K. Hills-Kimball, T. Cai, W. Shi, Z. Wei, H. Yang, Y. Candler, P. Wang, J. He, O. Chen, Stereoselective C–C oxidative coupling reactions photocatalyzed by zwitterionic ligand capped $CsPbBr_3$ perovskite quantum dots. *Angew. Chem. Int. Ed.* **132**, 22752–22758 (2020).

43. F. Zhang, H. Zhong, C. Chen, X.-g. Wu, X. Hu, H. Huang, J. Han, B. Zou, Y. Dong, Brightly luminescent and color-tunable colloidal $CH_3NH_3PbX_3$ (X = Br, I, Cl) quantum dots: Potential alternatives for display technology. *ACS Nano* **9**, 4533–4542 (2015).

44. H. Zhao, H. Chen, S. Bai, C. Kuang, X. Luo, P. Teng, C. Yin, P. Zeng, L. Hou, Y. Yang, L. Duan, F. Gao, M. Liu, High-brightness perovskite light-emitting diodes based on $FAPbBr_3$ nanocrystals with rationally designed aromatic ligands. *ACS Energy Lett.* **6**, 2395–2403 (2021).

45. G. Nedelcu, L. Protesescu, S. Yakunin, M. I. Bodnarchuk, M. J. Grotevent, M. V. Kovalenko, Fast anion-exchange in highly luminescent nanocrystals of cesium lead halide perovskites ($CsPbX_3$, X = Cl, Br, I). *Nano Lett.* **15**, 5635–5640 (2015).

46. J. Almutlaq, W. J. Mir, L. Gutierrez-Arzaluz, J. Yin, S. Vasylevskyi, P. Maity, J. Liu, R. Naphade, O. F. Mohammed, O. M. Bakr, $CsMnBr_3$: Lead-free nanocrystals with high photoluminescence quantum yield and picosecond radiative lifetime. *ACS Materials Lett.* **3**, 290–297 (2021).

47. F. Krieg, S. T. Ochsenbein, S. Yakunin, S. ten Brinck, P. Aellen, A. Suess, B. Clerc, D. Guggisberg, O. Nazarenko, Y. Shynkarenko, S. Kumar, C.-J. Shih, I. Infante, M. V. Kovalenko, Colloidal $CsPbX_3$ (X = Cl, Br, I) nanocrystals 2.0: Zwitterionic capping ligands for improved durability and stability. *ACS Energy Lett.* **3**, 641–646 (2018).

48. F. Di Stasio, S. Christodoulou, N. Huo, G. Konstantatos, Near-unity photoluminescence quantum yield in $CsPbBr_3$ nanocrystal solid-state films via postsynthesis treatment with lead bromide. *Chem. Mater.* **29**, 7663–7667 (2017).

49. P. Du, J. Li, L. Wang, L. Sun, X. Wang, X. Xu, L. Yang, J. Pang, W. Liang, J. Luo, Y. Ma, J. Tang, Efficient and large-area all vacuum-deposited perovskite light-emitting diodes via spatial confinement. *Nat. Commun.* **12**, 4751 (2021).

50. L. M. Wheeler, E. M. Sanehira, A. R. Marshall, P. Schulz, M. Suri, N. C. Anderson, J. A. Christians, D. Nordlund, D. Sokaras, T. Kroll, S. P. Harvey, J. J. Berry, L. Y. Lin, J. M. Luther, Targeted ligand-exchange chemistry on cesium lead halide perovskite quantum dots for high-efficiency photovoltaics. *J. Am. Chem. Soc.* **140**, 10504–10513 (2018).

51. Y.-Q. Zheng, Y. Liu, D. Zhong, S. Nikzad, S. Liu, Z. Yu, D. Liu, H.-C. Wu, C. Zhu, J. Li, H. Tran, J. B.-H. Tok, Z. Bao, Monolithic optical microlithography of high-density elastic circuits. *Science* **373**, 88–94 (2021).

52. Y. Hassan, J. H. Park, M. L. Crawford, A. Sadhanala, J. Lee, J. C. Sadighian, E. Mosconi, R. Shivanna, E. Radicchi, M. Jeong, C. Yang, H. Choi, S. H. Park, M. H. Song, F. D. Angelis, C. Y. Wong, R. H. Friend, B. R. Lee, H. J. Snaith, Ligand-engineered bandgap stability in mixed-halide perovskite LEDs. *Nature* **591**, 72–77 (2021).

53. M. Imran, V. Caligiuri, M. Wang, L. Goldoni, M. Prato, R. Krahne, L. De Trizio, L. Manna, Benzoyl halides as alternative precursors for the colloidal synthesis of lead-based halide perovskite nanocrystals. *J. Am. Chem. Soc.* **140**, 2656–2664 (2018).

54. J.-N. Yang, Y. Song, J.-S. Yao, K.-H. Wang, J.-J. Wang, B.-S. Zhu, M.-M. Yao, S. U. Rahman, Y.-F. Lan, F.-J. Fan, H.-B. Yao, Potassium bromide surface passivation on $CsPbI_{3-x}Br_x$ nanocrystals for efficient and stable pure red perovskite light-emitting diodes. *J. Am. Chem. Soc.* **142**, 2956–2967 (2020).

55. D. W. Y. Teo, Z. Jamal, Q.-J. Seah, R.-Q. Png, L.-L. Chua, General bis(fluorophenyl azide) photo-crosslinkers for conjugated and non-conjugated polyelectrolytes. *J. Mater. Chem. C* **8**, 253–261 (2020).

56. Q. A. Akkerman, M. Gandini, F. Di Stasio, P. Rastogi, F. Palazon, G. Bertoni, J. M. Ball, M. Prato, A. Petrozza, L. Manna, Strongly emissive perovskite nanocrystal inks for high-voltage solar cells. *Nat. Energy* **2**, 16194 (2017).

57. C. Zou, C. Chang, D. Sun, K. F. Böhringer, L. Y. Lin, Photolithographic patterning of perovskite thin films for multicolor display applications. *Nano Lett.* **20**, 3710–3717 (2020).

58. J. Mao, W. E. Sha, H. Zhang, X. Ren, J. Zhuang, V. A. Roy, K. S. Wong, W. C. Choy, Novel direct nanopatterning approach to fabricate periodically nanostructured perovskite for optoelectronic applications. *Adv. Funct. Mater.* **27**, 1606525 (2017).

59. S. Ma, S. H. Kim, B. Jeong, H. C. Kwon, S. C. Yun, G. Jang, H. Yang, C. Park, D. Lee, J. Moon, Strain-mediated phase stabilization: A new strategy for ultrastable $\alpha$-$CsPbI_3$ perovskite by nanoconfined growth. *Small* **15**, 1900219 (2019).

60. Y. Lei, Y. Chen, Y. Gu, C. Wang, Z. Huang, H. Qian, J. Nie, G. Hollett, W. Choi, Y. Yu, N. Kim, C. Wang, T. Zhang, H. Hu, Y. Zhang, X. Li, Y. Li, W. Shi, Z. Liu, M. J. Sailor, L. Dong, Y. Lo, J. Luo, S. Xu, Controlled homoepitaxial growth of hybrid perovskites. *Adv. Mater.* **30**, 1705992 (2018).

61. M. Duan, Z. Feng, Y. Wu, Y. Yin, Z. Hu, W. Peng, D. Li, S.-j. Chen, C.-Y. Lee, A. Lien, Inkjet-printed micrometer-thick patterned perovskite quantum dot films for efficient blue-to-green photoconversion. *Adv. Mater. Technol.* **4**, 1900779 (2019).

62. M. Zhu, Y. Duan, N. Liu, H. Li, J. Li, P. Du, Z. Tan, G. Niu, L. Gao, Y. Huang, Z. Yin, J. Tang, Electrohydrodynamically printed high-resolution full-color hybrid perovskites. *Adv. Funct. Mater.* **29**, 1903294 (2019).
